# Heterochromatin protein 1 alpha (HP1α) undergoes a monomer to dimer transition that opens and compacts live cell genome architecture

**DOI:** 10.1093/nar/gkae720

**Published:** 2024-08-28

**Authors:** Jieqiong Lou, Qiji Deng, Xiaomeng Zhang, Charles C Bell, Andrew B Das, Naiara G Bediaga, Courtney O Zlatic, Timothy M Johanson, Rhys S Allan, Michael D W Griffin, PrasadN Paradkar, Kieran F Harvey, Mark A Dawson, Elizabeth Hinde

**Affiliations:** School of Physics, University of Melbourne, Melbourne, VIC 3010, Australia; Peter MacCallum Cancer Centre, 305 Grattan St, Melbourne, VIC 3000, Australia; School of Physics, University of Melbourne, Melbourne, VIC 3010, Australia; Peter MacCallum Cancer Centre, 305 Grattan St, Melbourne, VIC 3000, Australia; Peter MacCallum Cancer Centre, 305 Grattan St, Melbourne, VIC 3000, Australia; Sir Peter MacCallum Department of Oncology, University of Melbourne, Parkville, VIC 3010, Australia; Peter MacCallum Cancer Centre, 305 Grattan St, Melbourne, VIC 3000, Australia; Department of Biochemistry and Pharmacology, Bio21 Molecular Science and Biotechnology Institute, University of Melbourne, Melbourne, VIC 3010, Australia; The Walter and Eliza Hall Institute of Medical Research, Parkville, VIC 3052, Australia; Department of Medical Biology, The University of Melbourne, Parkville, VIC 3010, Australia; The Walter and Eliza Hall Institute of Medical Research, Parkville, VIC 3052, Australia; Department of Medical Biology, The University of Melbourne, Parkville, VIC 3010, Australia; Department of Biochemistry and Pharmacology, Bio21 Molecular Science and Biotechnology Institute, University of Melbourne, Melbourne, VIC 3010, Australia; CSIRO Health & Biosecurity, Australian Centre for Disease Preparedness, 5 Portarlington Road, Geelong3220, Australia; Peter MacCallum Cancer Centre, 305 Grattan St, Melbourne, VIC 3000, Australia; Sir Peter MacCallum Department of Oncology, University of Melbourne, Parkville, VIC 3010, Australia; Department of Anatomy and Developmental Biology and Biomedicine Discovery Institute, Monash University, Clayton, VIC 3168, Australia; Peter MacCallum Cancer Centre, 305 Grattan St, Melbourne, VIC 3000, Australia; Sir Peter MacCallum Department of Oncology, University of Melbourne, Parkville, VIC 3010, Australia; Centre for Cancer Research, University of Melbourne, Melbourne, VIC 3010, Australia; School of Physics, University of Melbourne, Melbourne, VIC 3010, Australia; Department of Biochemistry and Pharmacology, Bio21 Molecular Science and Biotechnology Institute, University of Melbourne, Melbourne, VIC 3010, Australia

## Abstract

Our understanding of heterochromatin nanostructure and its capacity to mediate gene silencing in a living cell has been prevented by the diffraction limit of optical microscopy. Thus, here to overcome this technical hurdle, and directly measure the nucleosome arrangement that underpins this dense chromatin state, we coupled fluorescence lifetime imaging microscopy (FLIM) of Förster resonance energy transfer (FRET) between histones core to the nucleosome, with molecular editing of heterochromatin protein 1 alpha (HP1α). Intriguingly, this super-resolved readout of nanoscale chromatin structure, alongside fluorescence fluctuation spectroscopy (FFS) and FLIM-FRET analysis of HP1α protein-protein interaction, revealed nucleosome arrangement to be differentially regulated by HP1α oligomeric state. Specifically, we found HP1α monomers to impart a previously undescribed global nucleosome spacing throughout genome architecture that is mediated by trimethylation on lysine 9 of histone H3 (H3K9me3) and locally reduced upon HP1α dimerisation. Collectively, these results demonstrate HP1α to impart a dual action on chromatin that increases the dynamic range of nucleosome proximity. We anticipate that this finding will have important implications for our understanding of how live cell heterochromatin structure regulates genome function.

## Graphical Abstract

**Graphical Abstract:**
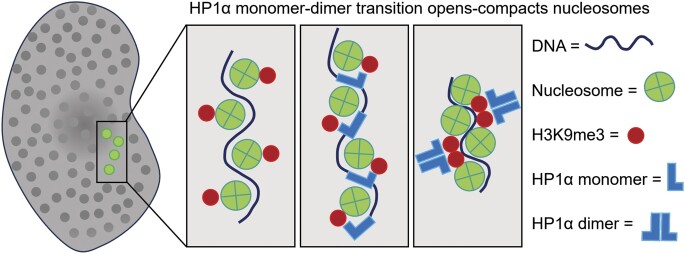

## Introduction

Inside the nucleus of a living human cell, approximately 2 m of DNA is wrapped around histone proteins to form a string of nucleosomes, and the resulting chromatin fibres are compacted into a three-dimensional (3D) chromatin network that occupies a ten-micrometre diameter nuclear volume. At any moment in time, this 3D architectural framework is undergoing local rearrangements in nucleosome proximity along each chromatin fibre, which update the DNA sequences available to the proteins that read and copy genetic information (1,2). These nanoscale structural dynamics lead to a continuously evolving nuclear landscape that is underpinned by a spectrum of chromatin states, which lie between the more open and biologically active euchromatin, versus the more dense and transcriptionally repressed heterochromatin state (3,4). In the case of heterochromatin, a multivalent architectural protein called heterochromatin protein 1 alpha (HP1α) is known to establish and maintain this chromatin state by: (1) binding to nucleosomes that exhibit trimethylation on lysine 9 of histone H3 (H3K9me3) (5–8), (2) self-associating into HP1α dimers that bridge together nucleosomes (9–11), and (3) condensing chromatin into globular nuclear structures that locally silence gene expression (12–16). While the mechanism behind HP1α dimer stacking of H3K9me3 marked nucleosomes has been directly observed *in vitro* (10,11,17), the impact of HP1α dimerisation on nucleosome proximity throughout chromatin in an intact nucleus remains unknown because the nucleosomal sub-unit is well below the diffraction limit of optical microscopy (18–20). Addressing this research gap and uncovering the role of HP1α self-association in maintenance of nucleus wide chromatin architecture, thus requires access to a super-resolved readout of live cell chromatin structure.

HP1α is underpinned by a tripartite structure, where a N-terminal chromodomain (CD) connected via a flexible hinge region to a C-terminal chromoshadow domain (CSD), collectively facilitate HP1α recognition of H3K9me3 (5,6), interaction with nucleic acids (21), dimer formation (22,23), and interaction with other factors (24–27). Over the past ten years an extensive series of biochemical, biophysical, and structural studies, which employ single point mutations in both the HP1α CD and CSD domain, have demonstrated that HP1α chromatin binding dynamics are modulated by H3K9me3 density (28–30) on a timescale of milliseconds to seconds (11,31–34), and the HP1α dimer interface promotes prolonged binding to H3K9me3 modified chromatin (10,35,36). Since H3K9me3 modified chromatin is known from micrococcal nuclease (MNase) digestion and high throughput sequencing to be associated with high nucleosome occupancy (37), the expectation is that the heterochromatin state maintained by HP1α is underpinned by a highly compact internal chromatin structure. However, to what extent this expectation applies to heterochromatin in a living cell is unclear, because while HP1α molecules globally decorate the nuclear wide chromatin network via binding to H3K9me3 (38), in different cellular contexts they also locally assemble chromatin into nuclear condensates that: (1) exhibit 3D liquid-liquid phase separated (LLPS) like properties (12–14,39), (2) behave as polymer globules percolated with nucleoplasmic liquid (40,41), and (3) are associated with a more open chromatin state (42). Thus, we set out to investigate the impact chromatin bound HP1α monomers versus dimers have on nucleosome proximity by fluorescence lifetime imaging microscopy (FLIM) of Förster resonance energy transfer (FRET) between fluorescently labelled core histones in live human cell nuclei and map the nanostructure of live cell higher order chromatin structure.

FRET is an optical phenomenon exquisitely sensitive to the distance between fluorophores that can report nucleosome spacing (within and or between chromatin fibres) on a scale of 1–10 nm in a living cell when the nucleosomes of the chromatin network are labelled with fluorescent donor and acceptor histones (43). The phasor approach to FLIM is a robust method of analysis for the detection of histone FRET (44,45), which can spatiotemporally map this nanoscale readout of chromatin compaction in a living cell in the presence of cellular autofluorescence (46,47), and upon fixation, with respect to epigenetic marks labelled via immunofluorescence (IF) (48,49). Here using phasor FLIM of histone FRET in human cells co-expressing histone H2B tagged to eGFP and mCherry, alongside a diffraction limited image analysis of DNA density (50,51) and stable expression of wild type HP1α versus a HP1α dimer mutant (I165E) (10,52), we find that HP1α self-association regulates sub-micron nuclear wide chromatin architecture via a two-step mechanism that occurs on a nanoscale. Specifically, we find that HP1α monomers, which are the dominant species according to fluorescence fluctuation spectroscopy (FFS) of HP1α tagged to eGFP, impart a global spacing between nucleosomes that is mediated by H3K9me3, and this spacer can be locally removed upon HP1α dimer formation. Given that nucleosome proximity is known to regulate DNA template access in a living cell, we anticipate that these nanoscale chromatin structure dynamics will have important implications for our understanding of how heterochromatin structure regulates key genomic functions such as transcription or DNA repair.

## Materials and methods

### Cloning

eGFP (Addgene, #54767), GFP-HP1α (Addgene, #17652), GFP-HP1β (Addgene, #17651), GFP-HP1γ (Addgene, #17650), SUV39H1 (Addgene, #82236), pEGFP-Trim28 (Addgene, #45568), KRAB (Addgene, #11642) and MeCP2 (Addgene, #48078) were purchased from Addgene. GFP-HP1α_I165E_ was a gift from Prof. Lori Wallrath's lab. pTagRFP657 and Cas9 were a gift from Prof. Katharina Gaus's lab. eGFP-HP1α and eGFP-HP1α_I165E_ were generated by PCR amplification of the HP1α and HP1α_I165E_ genes and insertion of them into the pEGFP-C1 vector with XhoI + BamHI double digestion and ligation. pTagRFP657 was amplified by primers 5′AGTAGTGCTAGCATGAGCGAGCTGATCACCGA3’ & 5′AGTAGTGAATTCATTAAGCTTGTGCCCCAGT3’ and cloned into the GFP-HP1α wild type and mutant vectors for replacing GFP between restriction enzyme NheI and EcoRI by standard digestion and ligation strategy. HP1α, HP1α_I165E_, eGFP-HP1α, and eGFP-HP1α_I165E_ vectors for stable transfection cell line establishment were generated by two steps: (1) IRES-GFP sequences from PB-Cuo-MCS-IRES-GFP-EF1α-CymR-Puro (cat. log. PBQM812A-1, Biosystem Sciences) were deleted by using 5′GCGGCCGCAGCTGAATCTAAGTCGACGG3’ and 5′ATTCAGCTGCGGCCGCGGATCCGATTTA3’, and (2) HP1α / HP1α_I165E_ were amplified with 5′AGTAGTGCTAGCATGGGAAAGAAAACCAAGCGGAC3’ and 5′AGTAGTGCGGCCGCTTAGCTCTTTGCTGTTTCTT3’ and cloned into PB-Cuo-MCS- EF1α-CymR-Puro between NheI and NotI, while eGFP-HP1α / eGFP-HP1α_I165E_ were shuttled from pEGFP-C1 to PB-Cuo-MCS-EF1α-CymR-Puro by NheI and NotI digestion. Two guide RNAs (gRNA) targeting the human *CBX5* gene exon 3 were designed and cloned into a gRNA expression vector with a U6 promotor by PCR overlapping extension (gRNA1 primers 5′AGTATAAGAAGATGAAGGGTTTTAGAGCTAG3’ & 5′TTCATCTTCTTATACTTTCGGTGTTTCGTCC3’; gRNA2 primers 5′ACTTTCTGACTTCTCCCTGTTTTAGAGCTAG3’ & 5′GGAGAAGTCAGAAAGTAACGGTGTTTCGTCC3’). SUV39H1-mCherry and KRAB-mCherry were generated by PCR amplification of the SUV39H1 and KRAB genes and insertion of them into the pmCherry-C1 vector by double digestion with KpnI + BamHI and ligation. To generate the plasmid RFP657-Trim28, RFP657 was amplified with PCR and inserted into pEGFP-TRIM28 by double digestion with NheI + BspEI to replace EGFP. MeCP2-RFP657 was made by PCR amplification of the MeCP2 gene and insertion into pTagRFP657 by double digestion with XhoI + BamHI and ligation. To express HP1α and eGFP-HP1α proteins in bacteria cells, pET28a-HP1α and eGFP-HP1α constructs were made by the following steps. A NheI restriction enzyme site was introduced to the pET28a vector by mutagenesis with the following primers: CAAGCTTGTCGACGGAGCTAGCAATTCGGATCCTGGC, GCCAGGATCCGAATTGCTAGCTCCGTCGACAAGCTTG; then HP1α and eGFP-HP1α were amplified by PCR and inserted into the pET28a vector with BamHI + NheI double digestion and ligation. All constructs were confirmed by Sanger sequencing (AGRF, Melbourne).

### Stable HeLa cell-line generation and transient knock down of HP1α expression

The eGFP-HP1α, eGFP-HP1α_I165E_, and HP1α_I165E_ Piggybac transposon vectors PB-Cuo-eGFP-HP1α-EF1α-CymR-Puro, PB-Cuo-eGFP-HP1αI165E-EF1α-CymR-Puro, and PB-Cuo-HP1αI165E-EF1α-CymR-Puro were transfected into wild type HeLa with the Super piggyBac Transposase expression vector (cat. log. PB210PA-1, System Biosciences) and use of Lipofectamine 3000 according to the manufacturer's protocol. Transfected HeLa cells were then cultured in the presence of 2 μg/ml puromycin for 2 weeks and the puromycin resistant cells propagated to generate HeLa stably transfected with eGFP-HP1α (HeLa^eGFP-HP1α^), eGFP-HP1α_I165E_ (HeLa^eGFP-HP1αI165E^) and HP1α_I165E_ (HeLa^HP1αI165E^). Selective siRNA knockdown (KD) of endogenous HP1α in wild type HeLa (HeLa^KD^) and the cell lines exogenously transfected with eGFP-HP1α, eGFP-HP1α_I165E_ or HP1α_I165E_ (HeLa^eGFP-HP1α+KD^, HeLa^eGFP-HP1αI165E+KD^, and HeLa^HP1αI165E+KD^) was achieved via transfection of three siRNA duplexes designed to target the mRNA 3′ untranslated region (3′UTR) of HP1α with a sense strand sequence of 5′GUUGGAAUCUUACUAGUC3’, 5′CUGACAUGUUGAGAUGG3’, and 5′CUUCUGUAAAGUGAUAUC3’ using Lipofectamine RNAiMAX (ThermoFisher) according to the manufacturer's protocol. HP1α immunofluorescence (IF) was employed to validate endogenous HP1α KD efficiency (Supplementary Figures S1 and S2) and exogenous HP1α expression (Supplementary Figure S3) in HeLa and HeLa^HP1αI65E^ versus HeLa^eGFP-HP1α^ and HeLa^eGFP-HP1αI165E^ pretreated with 100 μg/ml cycloheximide 3 h before cell fixation to minimise the impact of non-mature eGFP (53). For imaging experiments that required co-transfection with H2B-eGFP, H2B-mCherry, RFP657-HP1α, RFP657-HP1α_I165E_, SUV39H1-mCherry, KRAB-mCherry, RFP657-Trim28, and MeCP2-RFP657, these cell lines were transfected 24 h post siRNA transfection using Lipofectamine 3000, and imaging measurements were conducted 48 h post siRNA transfection.

### Quantification of exogenous HP1α expression levels

As mentioned above, HP1α IF was employed to quantify HP1α exogenous expression in HeLa, HeLa^eGFP-HP1α^, HeLa^eGFP-HP1αI165E^ and HeLa^HP1αI165E^ treated with scrambled siRNA (control) versus siRNA KD targeting HP1α 3′UTR for 48 h alongside cycloheximide for 3 h, to generate HeLa^KD^, HeLa^eGFP-HP1α+KD^, HeLa^eGFP-HP1αI165E+KD^ and HeLa^HP1αI165E+KD^. The intensity of eGFP and HP1α IF were plotted to determine the correlation between eGFP signal and HP1α IF intensity, and guide selection of cells for microscopy experiments that express eGFP-HP1α and eGFP-HP1α_I165E_ in an equivalent amount to endogenous HP1α. For HeLa^HP1αI165E^ cells, two colonies C2 and C5, with slightly lower (85–95%) HP1α_I165E_ expression level compared to endogenous HP1α, were generated, and clone C2 was selected for microscopy experiments since C2 had less variable HP1α_I165E_ expression (Supplementary Figure S2).

### CRISPR/Cas9 HP1α knock out HeLa cell line establishment and characterisation

HeLa were simultaneously transfected with two *CBX5* targeted gRNA vectors and the Cas9 expression vector by Lipofectamine 3000 according to the manufacturer's protocol. Twenty-four hours after transfection, cells were counted, diluted and an average of 1 cell per well was plated into a 96 well plate. Cells were cultured in a 96-well plate for two weeks, with 50 μl of fresh medium added to each well every 4 days. Single cell colonies were expanded to approximately 1–2 million cells, then a portion of the colonies were screened by western blot (WB) to verify HP1α knock out (KO) (Cat. 05–689, Millipore). This involved cells being pelleted and lysed by Ringer's lysis buffer, and the cell lysate being centrifuged at 15000 g for 10 minutes at 4°C. The protein concentration in the supernatant was then quantified by the BCA assay and a total of 20 μg denatured protein loaded into each well of a homemade 12.5% Sodium dodecyl-sulfate polyacrylamide gel. Proteins were separated by application of 150 V for 1 h. Proteins were transferred to nitrocellulose membrane at 35 V for 2.5 h on ice. Membranes were blocked by 5% bovine serum albumin (BSA) for 1 h at room temperature and incubated with primary antibody at 4°C overnight. Membranes were washed with PBS-Tween 20 (PBS-T) 3 times and incubated with HRP conjugated secondary antibodies for 1 h at room temperature, after which the membrane was then washed with PBS-T 3 times, incubated with ECL western blotting substrate and imaged by a luminescent image analyzer (LAS-4000, GE Healthcare). After WB verification, genomic DNA was extracted from potential HP1α KO colonies, the HP1α gene segment was amplified by PCR and sequenced for further KO verification. One cell colony, colony 2F8, was selected for HeLa HP1α KO imaging experiments (HeLa^HP1α-KO^) and stably transfected with HP1α (HeLa^HP1α-KO+HP1α^) and HP1α_I165E_(HeLa^HP1α-KO+HP1αI165E^) (Supplementary Figure S4), using the Piggybac transposon system previously described for generation of HeLa^eGFP-HP1α^, HeLa^eGFP-HP1αI165E^, and HeLa^HP1αI165E^.

### Cell culture, transient transfection, immunofluorescence and DNA staining

HeLa and HeLa^HP1α-KO^ cells were grown in DMEM (Lonza) supplemented with 10% bovine growth serum (Gibco), 1 × Pen-Strep (Lonza) at 37°C in 5% CO_2_. HeLa^eGFP-HP1α^, HeLa^eGFP-HP1αI165E^, HeLa^HP1αI165E^, HeLa^HP1α-KO+HP1α^ and HeLa^HP1α-KO+HP1αI165E^ cells were grown in DMEM (Lonza) supplemented with 10% bovine growth serum (Gibco), 1 × Pen-Strep (Lonza) and 2 μg/ml puromycin at 37°C in 5% CO_2_. For live cell microscopy experiments, cells were plated 48 h before experiments onto 35 mm glass bottom dishes and transiently transfected or co-transfected with plasmids (specified in each figure) 24 h before experiments via use of Lipofectamine 3000 according to the manufacturer's protocol. For immunofluorescence (IF) against HP1α (Cat. 05-689, Millipore) and H3K9me3 (Cat. ab8898, Abcam), cells were fixed with 4% paraformaldehyde for 15 minutes, permeabilized with 1 mg/ml Triton X-100 for 15 min at room temperature and blocked with 1% BSA, each in a PBS buffer. Primary antibody (1:250 dilution) was incubated overnight at 4 ºC. Secondary antibody (Cat. A21244, Invitrogen 1:1000) was incubated for 1 hr at room temperature. For DNA staining, cells were incubated with 1 μM of Hoechst 33342 for 10 min at 37°C.

### MNase digestion

Cells were trypsinised and collected in cold PBS then washed twice with PBS and resuspended in cold lysis buffer on ice for 5 min. Cells were spun down at 1400 rpm for 3 min and washed twice with washing buffer to collect nuclei. The nuclei were resuspended with MNase digestion buffer (54) and divided into multiple tubes with 1 million nuclei / tube. 0.5 unit of MNase (Sigma, N3755) was added into each of the tubes and DNA was digested for 2, 6 or 12 min. Stopping buffer was added and gently mixed. RNaseA was added and incubated at 37ºC for 1 h followed by Proteinase K incubation at 50ºC for 1 h. DNA was purified by phenol-chloroform extraction and ethanol precipitation. 2 μg of undigested and MNase digested DNA from each sample was loaded and run on 1.8 % agarose gel (37).

### Protein purification

BL21-CodonPlus (DE3)-RIL competent cells were transformed with HP1α or eGFP-HP1α plasmids and inoculated in 5 ml of LB-ampicillin media. The bacteria culture was first allowed to grow overnight (37ºC, 220 rpm) and then back diluted (1:100) to 250 ml of fresh LB supplemented with ampicillin and allowed to grow for 3 h (37ºC, 220 rpm). Subsequently, the cells were induced with 1 mM IPTG for 3 to 4 h (37 ºC, 220 rpm). The bacteria cells were pelleted at 3000 g for 15 min at room temperature and resuspended in 5 ml of lysis buffer containing 1× Bugbuster protein extraction reagent (Millipore, 70921), 20 mM Tris pH8.0, 250 mM KCl, 10% glycerol, 10 mM Imidazole, 1 mM PMSF and a cOmpleteTM EDTA-free protease inhibitor tablet (Sigma 1183617001) for 20 min at room temperature. The cell lysate was clarified by spinning down cell debris at 16000 g for 15 min at room temperature. 250 μl of Ni-NTA agarose bead slurry (ThermoFisher, 88221) was pre-equilibrated with 2.5 ml of equilibration buffer containing Tris pH8.0, 250 mM KCl, 10% glycerol, and 10 mM Imidazole. The clarified supernatant was then loaded onto Ni-NTA agarose beads and incubated for 30 min at room temperature. The protein-bound Ni-NTA beads were washed with 5 ml of wash buffer containing Tris pH8.0, 250 mM KCl, 10% glycerol, 20 mM Imidazole, and 1 mM PMSF. Protein was eluted with 250 μl of elution buffer containing Tris pH 8.0, 250 mM KCl, 10% glycerol, 250 mM Imidazole, and 1 mM PMSF 3 times. The eluted fractions were tested on an SDS-PAGE gel and imaged by Coomassie blue. The protein was subsequently purified using a HiLoad 16/600 Superdex 75 pg size exclusion column with buffer containing 20 mM Tris pH8.0 and 250 mM potassium chloride.

### Absorbance-detected sedimentation velocity analytical ultracentrifugation

Sedimentation velocity analytical ultracentrifugation (SV-AUC) experiments were conducted using a Beckman Coulter Optima AUC analytical ultracentrifuge, equipped with UV-visible scanning optics. Reference (20 mM Tris, 250 mM potassium chloride, pH 8.0) and sample solutions were loaded into double-sector 12 mm cells with quartz windows and centrifuged using an An-50 Ti rotor at 50 000 rpm (201 600 g) and 20°C. Radial absorbance data were collected at 230 or 220 nm. Sedimentation data were fitted to a continuous sedimentation coefficient, c(s), model using SEDFIT (55) and converted to s20,w. Buffer density, viscosity, and partial specific volume of the protein samples were calculated using SEDNTERP (56). Sedimentation coefficient isotherms were generated by integration of c(s20,w) distributions between 1.0 S and 8.0 S, encompassing signal for monomer and dimer of both HP1α and eGFP-HP1α. Each isotherm was fitted to a monomer-dimer self-association model in SEDPHAT (57). 68% confidence intervals were estimated by projection of the error surface in SEDPHAT.

### *In situ* Hi-C

*In situ* Hi-C was performed as previously described (58). In brief, 0.5–5 million cells were used as input and restriction digestion was performed using Mbo1. Libraries were size selected between 200 and 1000 bp and sequenced on the NextSeq500 or 2000 with the paired end 75 bp chemistry. To visualise relative contact probability (RCP) across the genome we used the RCP function in GENome Organisation Visual Analytics (GENOVA) (59). In order to generate Hi-C matrices in the correct format for GENOVA, Hi-C libraries were first aligned using BWA-MEM (60) and mcool matrices at 50–100 kb resolution were constructed and normalised using Hi-C explorer (61). All Hi-C data has been deposited into the Gene Expression Omnibus (GEO).

### Fluorescence fluctuation spectroscopy (FFS)

All FFS measurements for number and brightness (NB) analysis, raster image correlation spectroscopy (RICS) and cross RICS, were performed on an Olympus FV3000 confocal laser scanning microscope coupled to an ISS A320 Fast FLIM box for fluorescence fluctuation data acquisition. For single channel NB FFS measurements, eGFP tagged plasmids were excited by a solid-state laser diode operating at 488 nm and the resulting fluorescence signal was directed through a 405/488/561 dichroic mirror to an external photomultiplier detector (H7422P-40 of Hamamatsu) fitted with an eGFP 500/25 nm bandwidth filter. For dual channel RICS FFS measurements (that enable cross RICS), the eGFP and mCherry (or RFP657) plasmid combination, were excited by solid-state laser diodes operating at 488 and 561 nm (or 640 nm) (respectively), and the resulting signal was directed through a 405/488/561/640 dichroic mirror to two internal GaAsP photomultiplier detectors set to collect 500–540 and 620–720 nm (or 650–750 nm) (respectively).

All FFS data acquisitions (i.e. NB, RICS and cross RICS) employed a 60X water immersion objective (1.2 NA) and first involved selecting a 10.6 μm region of interest (ROI) within a HeLa cell nucleus at 37 degrees in 5% CO_2_ that exhibited eGFP expression at an endogenous level; and in the case of dual channel FFS acquisitions, a nanomolar co-expression of mCherry or RFP657; which collectively ensured observation of fluctuations in fluorescence intensity (62,63). Then a single or simultaneous two channel frame scan acquisition was acquired (*N* = 100 frames) in the selected ROI with a pixel frame size of 256 × 256 (i.e. pixel size ∼ 41 nm) and a pixel dwell time of 12.5 μs. These conditions resulted in a scanning pattern that was found to be optimal for simultaneous capture of the apparent brightness and mobility of the different eGFP, mCherry and RFP657 constructs being characterised by NB, RICS and cross RICS analysis; all of which was performed in the SimFCS software developed at the Laboratory for Fluorescence Dynamics (LFD).

### Fluorescence lifetime imaging microscopy (FLIM)

All FLIM measurements for detection of Förster resonance energy transfer (FRET) were performed on an Olympus FV3000 confocal laser scanning microscope coupled to a 488 nm pulsed laser operated at 80 MHz and an ISS A320 Fast FLIM box for fluorescence lifetime data acquisition. This external laser line enabled selective excitation of the eGFP tagged plasmids that serve as a donor in each FRET experiment, and the FastFLIM box data acquisition card enabled changes in the donor's fluorescence lifetime to be extracted from eGFP’s fluorescent signal, which was directed through a 405/488/561 dichroic mirror to an external photomultiplier detector (H7422P-40, Hamamatsu) fitted with an eGFP 500/25 nm bandwidth filter. In each FRET experiment, the acceptor was an mCherry or RFP657 tagged plasmid, and in either case, prior to FLIM measurement of the donor (i.e. eGFP tagged plasmid), a dual channel intensity image of the acceptor-donor ratio was acquired using the same laser, dichroic and detectors settings described for cross RICS.

All FLIM data acquisitions of FRET employed a 60X water immersion objective (1.2 NA) and first involved selecting a 21 μm ROI across a HeLa cell nucleus at 37 degrees in 5% CO2 that exhibited co-expression of eGFP and mCherry or RFP657 with a high acceptor–donor ratio (i.e. > 1); a condition known to ensure the potential for detection of FRET (45). Then a single channel fluorescence lifetime data acquisition was integrated (*N* = 20 frames) in the selected ROI with a pixel frame size of 256 × 256 (i.e. pixel size ∼82 nm) and pixel dwell time of 20 μs via use of the ISS VistaVision software that pre-calibrates the instrument and phasor space against a known reference lifetime; we used fluorescein at pH 9 that has a single exponential lifetime of ∼4 ns. Phasor based FRET analysis of acquired FLIM data was performed in the SimFCS software developed at the Laboratory for Fluorescence Dynamics (LFD).

### Confocal laser scanning microscopy of Hoechst 33342 and immunofluorescence (IF)

All microscopy measurements recording Hoechst 33342 and IF intensity were performed on an Olympus FV3000 confocal laser scanning microscope. For the coefficient of variation (CV) analysis of DNA intensity images, Hoechst33342 was excited by a solid-state laser diode operating at 405 nm, and the resulting signal was directed through a 405/488/561 dichroic mirror to an internal GaAsP photomultiplier detector set to collect 430–470 nm. For dual channel IF measurements, the Alexa Fluorophore 405 (AF405) and 647 (AF647) combination, were excited by solid-state laser diodes operating at 405 nm and 640 nm (respectively), and the resulting signal was directed through a 405/488/561/640 dichroic mirror to two internal GaAsP photomultiplier detectors set to collect 430–470 and 650–750 nm (respectively).

All intensity image data acquisitions (i.e. CV and IF) employed a 60× water immersion objective (1.2 NA) and involved either: (1) a single channel frame scan acquisition (i.e. *N* = 1 frame) for CV analysis of a 21 μm ROI across a HeLa cell nucleus at 37 degrees in 5% CO_2_ with a pixel frame size of 256 × 256 (i.e. pixel size ∼82 nm) and a pixel dwell time of 12.5 μs, or (2) a dual channel frame scan acquisition (i.e. *N* = 1 frame) for IF quantification of a 21 μm ROI (or whole field of view) across a HeLa cell nucleus (or multiple nuclei) at room temperature with a pixel frame size of 256 × 256 (or 2048 × 2048) (i.e. pixel size ∼82 nm in either case) and a pixel dwell time of 12.5 μs. Both CV analysis of DNA density and image analysis of IF was performed in Fiji (ImageJ).

### Number and brightness (NB) analysis

The oligomeric state of the different eGFP-tagged plasmids investigated (i.e. eGFP-HP1α and eGFP-HP1α_I165E_) was extracted and spatially mapped throughout single channel FFS measurements via a moment-based brightness analysis that has been described in previously published papers (15,64). In brief, within each pixel of an NB FFS measurement there is an intensity fluctuation ${\mathrm{F}}( {\mathrm{t}} )$ which has: (i) an average intensity $\langle {{\mathrm{F}}( {\mathrm{t}} )} \rangle$ (first moment) and (ii) variance ${\sigma ^2}$ (second moment); and the ratio of these two properties describes the apparent brightness (B) of the molecules that give rise to the intensity fluctuation. The true molecular brightness (*ε*) of the fluorescent molecules being measured is related to B by ${\mathrm{B}} = {\mathrm{\varepsilon }} + 1$, where 1 is the brightness contribution of a photon counting detector. Thus, if we measure the B of monomeric eGFP (B_monomer_= *ε*_monomer_ + 1) under our NB FFS measurement conditions, then we can determine *ε*_monomer_ and extrapolate the expected B of eGFP-tagged dimers (B_dimer_= (2 × *ε*_monomer_) + 1) or oligomers (e.g. B_tetramer_= (4 × *ε*_monomer_) + 1), and in turn define brightness cursors, to extract and spatially map the fraction of pixels within a NB FFS measurement that contain these different species. These cursors were used to extract the fraction of eGFP-HP1α dimer and oligomer (i.e. number of pixels assigned B_dimer_ or B_oligomer_) within a NB FFS measurement and quantify the degree of HP1α self-association across multiple cells. Artefact due to cell movement or photobleaching were subtracted from acquired intensity fluctuations via use of a moving average algorithm and all brightness analysis was carried out in SimFCS from the Laboratory for Fluorescence Dynamics (LFD).

### RICS and ccRICS analysis

The concentration, mobility, and fraction of interaction between the different eGFP and mCherry or RFP657-tagged plasmids investigated was extracted via application of the RICS and cross RICS functions described in previously published papers to the dual channel FFS measurements (62,65,66). In brief, the fluorescence intensity recorded within each frame (N = 100) of each channel (i.e. CH1 and CH2) was spatially correlated via application of the RICS function, and spatially cross-correlated between channels (CC) via application of the cross RICS function, alongside a moving average algorithm (*N* = 10 frames) in both instances. Then the recovered RICS and cross RICS correlation profiles were fit to a 3D diffusion model and the amplitude versus decay of each fit recorded in the form of a G value and diffusion coefficient (*D*) respectively. The ratio of the cross RICS amplitude (i.e. G_CC_) with the limiting channel RICS amplitude (i.e. G_CH1_ or G_CH2_) enabled the fraction of eGFP-HP1α_I165E_ molecules interacting with mCherry or RFP657 tagged HPα, HP1β, HP1γ, SUV39H1, KRAB, MeCP2 or TRIM28 molecules to be extracted, while the RICS and cross RICS *D* values enabled changes in mobility to be detected. All RICS and cross RICS analysis was carried out in SimFCS from the Laboratory for Fluorescence Dynamics (LFD).

### FLIM-FRET analysis

Hetero FRET between the different eGFP (donor molecule) and mCherry or RFP657 (acceptor molecule) tagged plasmids investigated, was detected, and then spatially mapped throughout FLIM data by the phasor approach to lifetime analysis that has been described in previously published papers (44,45,48,67). In brief, the phasor approach first transforms the donor fluorescence lifetime recorded in each pixel of a FLIM image into a single point (a phasor) within a two-dimensional coordinate system (defined by parameters termed G and S), and then plots the resulting phasor distribution into a two-dimensional histogram called a phasor plot, where independent mixtures of fluorophores can be distinguished from changes in lifetime due to FRET. In pixels where donor molecules undergo FRET with acceptor molecules, the phasor coordinate is right shifted along a curved trajectory that is described by the classical definition of FRET efficiency. To determine the efficiency of the FRET state throughout FLIM data presented throughout the manuscript, the phasor coordinates of cellular autofluorescence versus the unquenched donor (i.e. eGFP-tagged plasmid in the absence of mCherry or RFP657-tagged plasmid) were first determined independently, and then a FRET trajectory was extrapolated from this baseline over the phasor distribution of the quenched donor (i.e., eGFP-tagged plasmid in the presence of mCherry or RFP657-tagged plasmid). From the FRET trajectory, coloured cursors were placed at the unquenched donor (i.e. 0% FRET) (teal cursor) versus quenched donor (e.g. 15% FRET efficiency) (red cursor) phasor locations and then the fraction as well as spatial distribution of FRET quantified and mapped across multiple cells. All phasor FLIM of FRET analysis was carried out in SimFCS from the Laboratory for Fluorescence Dynamics (LFD).

### Coefficient of variation (CV) analysis

The degree of spatial heterogeneity in DNA density throughout a single nucleus was quantified via a CV analysis of DNA intercalator Hoechst 33342, as described in previously published papers (50,51). In brief, this involved calculating the standard deviation in Hoechst 33342 fluorescence intensity throughout a frame scan image of a single nucleus and normalising this value to the mean fluorescence intensity to obtain a CV index. The CV index is directly proportional to the degree of sub-micron variation in Hoechst 33342 intensity and was thus used to monitor sub-micron changes in DNA density and chromatin structure. All CV analysis was performed in Fiji (ImageJ).

### Immunofluorescence (IF) quantification

IF intensity quantification of HeLa cell nuclei was performed by thresholding the Hoechst33342 signal in dual channel IF confocal images by Fiji(ImageJ) (68). The coordinates of the identified nuclei were then saved in the ImageJ region of interest (ROI) manager and the mean H3K9me3, eGFP-HP1α and HP1α IF intensity in the identified ROIs was calculated via use of ImageJ’s ‘measurement’ function.

### Statistics and figure preparation

Statistical analysis was performed by using GraphPad Prism software. Figures were prepared by use of Adobe Illustrator, ImageJ, and SimFCS.

## Results

### HP1α exists as a monomer and dimer in a living cell

**Figure 1.**
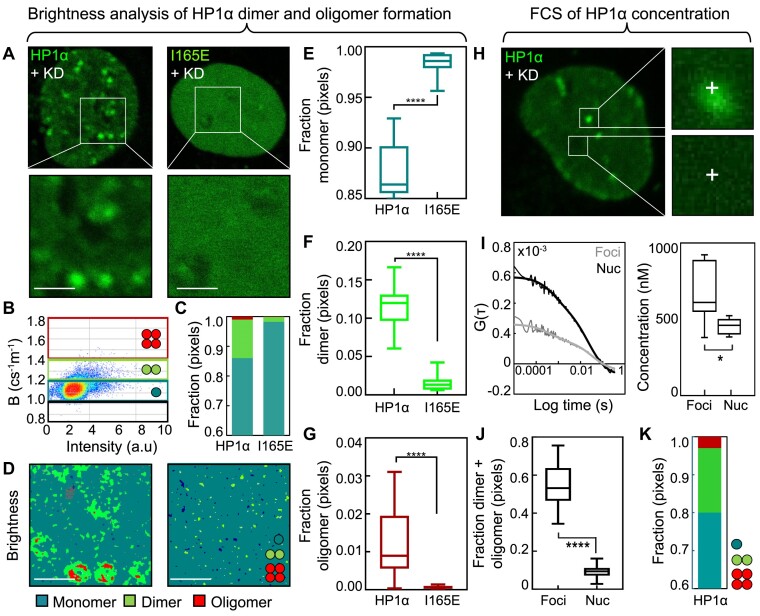
The majority of HP1α exists in a monomeric state in live cells. (**A**) Intensity images of the eGFP signal throughout a selected HeLa^eGFP-HP1α+KD^ versus HeLa^eGFP-HP1αI165E+KD^ cell (A, top) and the region of interest (ROI) from which each NB data acquisition was recorded (A, bottom). Scale bars 2 μm. (**B**) Intensity versus brightness scatterplot of the eGFP-HP1α NB data acquisition presented in (A) with the calibrated brightness windows superimposed (Supplementary Figure 6a–c). (**C**) Quantification of the fractional contribution of HP1α monomer (teal), dimer (green) and oligomer (red) in the NB data acquisitions presented in (A). (**D**) Brightness maps of the NB data acquisitions presented in (A) pseudo-coloured according to the brightness windows defined in (B) spatially map HP1α monomer (teal), dimer (green) and oligomer (red) localisation. (**E–G**) Quantification of the fraction of pixels containing monomer, dimer, and oligomer in HeLa^eGFP-HP1α+KD^ versus HeLa^eGFP-HP1αI165E+KD^ (N = 14 cells, two biological replicates). (**H**) Intensity image of the eGFP signal throughout a selected HeLa^eGFP-HP1α+KD^ cell (H, left) and representative ROIs from which single point FCS acquisitions were recorded (i.e. foci versus nucleoplasm) (H, right). (**I**) ACF profiles that result from temporal correlation of fluctuations in eGFP-HP1α fluorescence intensity within ROIs containing foci versus nucleoplasm that exhibit an amplitude at τ = 0 (i.e. G(0)) that is inversely proportional to the local number of molecules present (I, left) and enables quantification of the average eGFP-HP1α concentration in these two distinct environments (I, right) (N = 9 measurements, 4–5 cells, one biological replicate). (**J**) Quantification of the fraction of pixels containing eGFP-HP1α dimer and oligomer within ROIs containing foci versus nucleoplasm in the data underpinning (E–G). (**K**) Quantification of the nuclear wide concentration weighted fraction of eGFP-HP1α self-association in the data underpinning (E–G). The box and whisker plots in (E-G), (I) and (J) show the minimum, maximum, sample mean: * *P* < 0.05**** *P* < 0.0001, un-paired t-test.

First, we sought to define and modulate the relative fractions of HP1α that are monomeric, dimeric, and oligomeric in the nuclei of live human cells by fluorescence fluctuation spectroscopy (FFS) (69) and use of a HP1α dimer mutant that inhibits self-association (HP1α_I165E_) (52). To do this at a physiological level, we first generated HeLa cell lines stably expressing eGFP tagged to wild type HP1α (eGFP-HP1α) (HeLa^eGFP-HP1α^) versus HP1α_I165E_ (eGFP-HP1α_I165E_) (HeLa^eGFP-HP1αI165E^); a fluorescent construct that in the case of wild type HP1α does not significantly alter the monomer-dimer dissociation constant of HP1α (Supplementary Figure S5); and designed a small interfering RNA (siRNA) that could in both cases, selectively knock down (KD) endogenous untagged HP1α (Supplementary Figure S3). Then in untreated HeLa versus HeLa^eGFP-HP1α^ and HeLa^eGFP-HP1αI165E^ treated with siRNA against endogenous HP1α (i.e. HeLa^eGFP-HP1α+KD^ and HeLa^eGFP-HP1αI165E+KD^) and cycloheximide to maximise eGFP maturation (53), we performed immunofluorescence (IF) against HP1α (Supplementary Figure S3a) and used the intensity of this signal to establish a window of eGFP expression in HeLa^eGFP-HP1α+KD^ and HeLa^eGFP-HP1αI165E+KD^ that correlated with endogenous HP1α in HeLa (Supplementary Figure S3b–d). This window of eGFP expression guided the selection of HeLa^eGFP-HP1α+KD^ and HeLa^eGFP-HP1αI165E+KD^ nuclei for FFS-based analysis of HP1α self-association and inhibition (Figure 1A).

FFS involves the statistical analysis of fluctuations in fluorescent protein intensity that are recorded in each pixel of a live cell microscopy experiment. Here, we first used a specific type of FFS termed number and brightness (NB) (64) to extract and spatially map the oligomeric state of eGFP-HP1α versus eGFP-HP1α_I165E_ within each pixel of a time series of confocal microscopy frames (i.e. an FFS dataset) acquired across the nuclei of HeLa^eGFP-HP1α+KD^ and HeLa^eGFP-HP1αI165E+KD^. NB extracts this information via a moment-based analysis (70) of the apparent brightness (B) of eGFP-HP1α versus eGFP-HP1α_I165E_, which can be directly related to the oligomeric state of HP1α and HP1α_I165E_ when the monomeric B of eGFP is known (15). Thus, NB analysis of FFS datasets acquired within HeLa nuclei transiently transfected with eGFP was first performed to calibrate the monomeric B of eGFP (B_monomer_= 1.15 ± 0.05) (Supplementary Figure S6a–c). Then eGFP-calibrated brightness windows were extrapolated and validated against eGFP multimers (Supplementary Figure S6d–f, left panels), to detect eGFP dimer (B_dimer_ = 1.3 ± 0.10) and higher order oligomer formation (*B*_oligomer_ = 1.6 ± 0.20) within FFS experiments performed in HeLa^eGFP-HP1α+KD^ and HeLa^eGFP-HP1αI165E+KD^ (Figure 1B and Supplementary Figure S6d–f, right panels).

From NB analysis of independent eGFP-HP1α and eGFP-HP1α_I165E_ FFS experiments (Figure 1C), we found the eGFP-calibrated brightness windows to accurately detect HP1α dimer and higher order oligomer formation as well as inhibition (respectively) within a nuclear wide population of HP1α monomers (Figure 1D). In particular, from quantification of multiple nuclei (*N* = 14 cells) we found that: (i) approximately 13% of wild type HP1α self-associates into dimers (11.5 ± 0.8%) and oligomers (1.2 ± 0.2%) at an endogenous level (Figure 1E–G); a nuclear wide fraction that is spatially correlated with local HP1α concentration (i.e. HP1α foci) (Figure 1A, D, left) as well as globally modulated by eGFP-HP1α under versus over expression (Supplementary Figure S7); and (ii) as predicted by biochemical studies (52) the I165E mutation significantly inhibits both self-associating HP1α populations (Figure 1E–G); a NB result verified in a HeLa HP1α knock out cell line (HeLa^HP1α-KO^) (Supplementary Figure S4) transiently transfected with eGFP-HP1α or eGFP-HP1α_I165E_ (Supplementary Figure S6h) and orthogonally confirmed by FLIM analysis of FRET between HP1α in HeLa^eGFP-HP1α+KD^ versus HeLa^eGFP-HP1αI165E+KD^ co-transfected with HP1α versus HP1α_I165E_ tagged to a red fluorescent protein (RFP657) (i.e. RFP657-HP1α and RFP657-HP1α_I165E_) (respectively) (Supplementary Figure S8).

From visual inspection of the wild type HP1α NB result it is evident that the spatial heterogeneity in local HP1α concentration is a factor that needs to be considered when quantifying the nuclear wide fraction of HP1α self-association. Thus, we next employed another type of FFS termed single point fluorescence correlation spectroscopy (FCS) alongside a masked NB analysis (Supplementary Figure S9), and quantified the concentration weighted fraction of HP1α monomer, dimer, and oligomer formation across HeLa^eGFP-HP1α+KD^ nuclei. Here single point FCS involved temporal autocorrelation of single point FFS datasets acquired within HP1α foci versus the nucleoplasm of HeLa^eGFP-HP1α+KD^ (Figure 1H) and a fit based extraction of the resulting autocorrelation function (ACF) amplitudes (G_0_) that are inversely proportional to the number of eGFP-HP1α molecules present in these two environments (Figure 1I). These single point FCS experiments together with an eGFP-HP1α intensity mask analysis of the NB data revealed that: (i) in HP1α foci versus the nucleoplasm of HeLa^eGFP-HP1α+KD^ the respective concentration of eGFP-HP1α is 815 ± 62 nM versus 470 ± 28 nM, while the respective fraction of HP1α self-association is 54% versus 10% (Figure 1J), and (ii) the nuclear wide concentration weighted fraction of wild type HP1α self-association in HeLa^eGFP-HP1α+KD^ is approximately 20% and underpinned by dimer (16.9 ± 2.6%) as well as oligomer (2.9 ± 0.7%) formation (Figure 1K).

Thus, in live human cells we find that the majority of HP1α is monomeric (a fraction that could be engaged in a heterotypic interaction) while the ∼20% of HP1α that self-associates into a homodimer or homo-oligomer throughout the cell nucleus is abolished by the I165E mutation.

### HP1α monomers exhibit an antagonistic function to HP1α dimers in the maintenance of nuclear wide chromatin network architecture

The fluorescence intensity images reporting eGFP-HP1α versus eGFP-HP1α_I165E_ localisation throughout NB data (Figure 1A), and the accompanying quantification of HP1α self-association as a function of local HP1α concentration (Figure 1H–K), demonstrate in agreement with previous studies that the spatial distribution of HP1α, and its propensity to form foci, depends on the presence of HP1α dimers (12,15). Thus, to next investigate if and how these changes in HP1α localisation are associated with a disruption to maintenance of underlying chromatin architecture; given HP1α is a chromatin architectural protein; we applied a coefficient of variation (CV) analysis (50,51,71–73) to HeLa nuclei in the presence versus absence of HP1α monomers and or dimers. To do so, we again employed siRNA KD of endogenous HP1α (Supplementary Figure S1) and generated a HeLa cell line stably expressing un-tagged HP1α_I165E_ (HeLa^HP1αI165E^) at an endogenous level (Supplementary Figure S2), to enable CV analysis of untreated HeLa, versus, HeLa and HeLa^HP1αI165E^ treated with siRNA against HP1α (i.e. HeLa^KD^ and HeLa^HP1αI165E+KD^).

**Figure 2.**
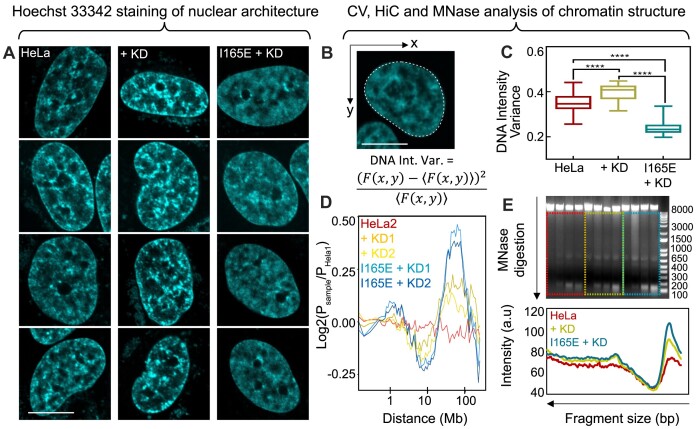
HP1α dimers induce sub-micron heterogeneity throughout a homogenous chromatin architecture that is maintained by HP1α monomers. (**A**) Intensity images of the Hoechst 33342 signal (i.e. DNA density) throughout the nuclei of HeLa (left), Hela^KD^ (middle), and HeLa^HP1αI165E+KD^ (right). Scale bar 10 μm. (**B**) Coefficient of variation (CV) analysis quantifies the heterogeneity in DNA density present throughout a Hoechst 33342 intensity image via calculation of the pixel intensity variance across the whole nucleus normalised to the pixel intensity mean (CV_index_). (**C**) Quantification of the CV_index_ across multiple HeLa, HeLa^KD^ and HeLa^HP1αI165E+KD^ nuclei (*N* ≥ 30 cells, two biological replicates). (**D**) Hi-C quantification of the frequency of short to long range chromatin interactions (0.1–100 Mb) throughout two replicates of Hela^KD^ and HeLa^HP1αI165E+KD^ (P_sample_) relative to HeLa (P_HeLa1_). (**E**) DNA fragmentation product (2 μg in each case) from MNase digestion of HeLa, HeLa^KD^, and HeLa^HP1αI165E+KD^ chromatin for an increasing amount of time (0, 2, 6 and 12 min at 0.5-unit MNase) (E, top panel), alongside quantification of the mean fluorescence intensity of the digested DNA fragments under the 12 min condition (E, bottom panel). The box and whisker plots in (C) show the minimum, maximum, sample mean: ****P< 0.0001, one-way ANOVA.

CV analysis scores heterogeneity in nuclear wide chromatin density on a sub-micron scale by calculation of the relative standard deviation in the fluorescence signal from a DNA intercalator such as Hoechst 33342 (50). Thus, to quantify the impact of HP1α monomers and or dimers on sub-micron chromatin architecture, we stained the nuclei of HeLa, HeLa^KD^ and HeLa^HP1αI165E+KD^ with Hoechst 33342 and then acquired confocal microscope images of the Hoechst 33342 fluorescence intensity (Figure 2A), to enable CV scoring of chromatin density (Figure 2B). Surprisingly, this analysis revealed that when HP1α monomers and dimers are both present in HeLa, there is significantly less heterogeneity in chromatin density (CV_index_ = 0.351 ± 0.008) to when they are absent in HeLa^KD^ (CV_index_ = 0.395 ± 0.006), and expression of only HP1α monomers in HeLa^HP1αI165E+KD^, resulted in a significantly more homogenous chromatin density phenotype (CV_index_ = 0.241 ± 0.006) than when HP1α dimers are present in HeLa (Figure 2C). This CV obtained result, which was verified in Hoechst 33342 stained HeLa^HP1α-KO^ nuclei transiently transfected with RFP657-HP1α or RFP657-HP1α_I165E_ (Supplementary Figure S10), collectively suggests HP1α monomers and dimers to be in opposition, and somehow, their net effect on chromatin structure leads to moderate chromatin condensation, on a sub-micron scale.

Next, to further investigate our CV-based quantitation of nuclear wide chromatin density, and identify, on what spatial scales the HP1α monomers and dimers are in opposition, we applied high-throughput chromosome conformation capture (Hi-C) (74) to the chromatin network of HeLa, HeLa^KD^ and HeLa^HP1αI165E+KD^, and quantified the frequency of short to long range chromatin interactions (0.1–100 Mb), which collectively occur, on up to a sub-micron scale (74). This involved proximity ligating the chromatin network of HeLa, HeLa^KD^ and HeLa^HP1αI165E+KD^, to generate genomic libraries that upon paired end sequencing, and data processing, produced contact frequency maps (58). Interestingly, analysis of these contact frequency maps relative to HeLa, revealed that the presence or absence of HP1α monomers and or dimers redistributes the frequency of short to long-range chromatin interactions (Figure 2D). Specifically, removal of HP1α promoted chromatin interactions into the 10–100 Mb range and the subsequent addition of HP1α monomers in HeLa^HP1αI165E+KD^ further promoted interaction in this range, whilst concurrently reducing interactions from the 0.1–1 Mb range (Figure 2D).

This Hi-C result alongside CV analysis suggests the spatial scale upon which HP1α monomers and dimers serve an antagonistic function in chromatin condensation is short range, and in the context of an intact nucleus this regulatory influence is potentially below the spatial resolution of sub-micron chromatin assessment. To orthogonally confirm this interpretation, and measure the impact of HP1α monomers and dimers on chromatin at a nanoscale (i.e. nucleosome proximity), we next digested the chromatin network of HeLa, HeLa^KD^ and HeLa^HP1αI165E+KD^ with MNase (an enzyme that cuts linker DNA between nucleosomes) and quantified the degree of DNA fragmentation in each case (37,54). This involved incubating HeLa, HeLa^KD^ and HeLa^HP1αI165E+KD^ with MNase and performing an intensity-based analysis of the DNA fragment product upon migration through agarose gel (Figure 2E and Supplementary Figure S11). Intriguingly, this MNase based analysis revealed the presence versus absence of HP1α dimers in HeLa and HeLa^HP1αI165E+KD^ to restrict versus promote DNA fragment production compared to HeLa^KD^. Thus, alongside Hi-C analysis, this MNase result confirms that *in vitro* HP1α monomer-dimer antagonism does occur down to the level of nucleosome proximity; a nanoscale feature of live cell chromatin structure.

### HP1α monomers impart a nanoscale nucleosome spacing throughout live cell chromatin network architecture that is locally reduced upon HP1α dimer formation

**Figure 3.**
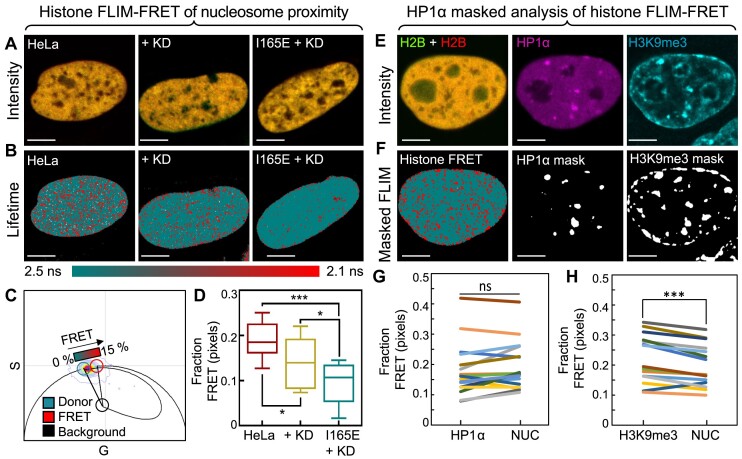
HP1α self-association is critical for nuclear wide chromatin network compaction while the HP1α monomeric subunit imparts a baseline level of de-compaction. (**A**) Merged intensity images of HeLa (A, left), HeLa^KD^ (A, middle) and HeLa^HP1αI165E+KD^ (A, right) nuclei expressing histone FRET reporter H2B-eGFP and H2B-mCh. Scale bars 5 μm. (**B-C**) Lifetime maps (B) of the FLIM data acquisitions presented in (A) pseudo-coloured according to the palette defined in the phasor plot in (C) spatially map open (teal) versus compact chromatin (red). Scale bars 5 μm. The phasor distribution of H2B-eGFP (C) detected throughout the lifetime maps in (B) is superimposed with a theoretical FRET trajectory that enables the efficiency of histone FRET with H2B-mCh (red cursor) to be characterised upon independent determination of the unquenched donor fluorescence lifetime (teal cursor) and cellular autofluorescence (black cursor) (Supplementary Figure S9a–d). (**D**) Quantification of the fraction of pixels exhibiting FRET (i.e. a compact chromatin state) across multiple HeLa, HeLa^KD^ and HeLa^HP1αI165E+KD^ nuclei (*N* ≥ 8 cells, two biological replicates.). (**E**) Intensity images of a HeLa nucleus co-expressing H2B-eGFP and H2B-mCh (E, left) with immunofluorescence (IF) against endogenous HP1α and histone modification H3K9me3 labelled with Alexa Fluorophore 647 (AF647) (E, middle) and AF405 (E, right) (respectively). Scale bars 5 μm. (**F**) Lifetime map (left) of the FLIM data acquisition presented in (E) pseudo-coloured according to no FRET (teal) versus FRET (red) alongside masks based on the HP1α-AF647 (F, middle) and H3K9me3-AF405 (F, right) intensity images presented in (E). Scale bars 5 μm. (**G-H**) Quantification of the fraction of histone FRET (compact chromatin) inside versus outside HP1α-AF647 (G) and H3K9me3-AF405 (H) intensity masks derived from multiple cells (*N* = 15 cells, two biological replicates). The box and whisker plot in (D) shows the minimum, maximum, sample mean: *P< 0.05, ***P< 0.001, one-way ANOVA. In (G-H): ns *P*> 0.05, ***P< 0.001, paired *t*-test.

CV analysis, Hi-C and MNase digestion, revealed HP1α monomers versus dimers to exhibit opposing functions in the maintenance of nuclear wide chromatin network architecture down to the level of nucleosome proximity (Figure 2A–E). To further dissect what this finding means in the context of live cell nuclear architecture, we next employed the phasor approach to FLIM for detection of FRET between fluorescently labelled histones core to the nucleosome, and spatially mapped the impact of HP1α self-association on nucleosome proximity, despite the diffraction limit of optical microscopy masking this nanoscale feature of chromatin structure (46). To do so, we employed a transient transfection of H2B tagged to eGFP (H2B-eGFP) and mCherry (H2B-mCh) as the histone FRET reporter in HeLa, HeLa^KD^, and HeLa^HP1αI165E+KD^ (Figure 3A).

Histone FRET reports nanoscale chromatin structure because the efficiency of energy transfer between the donor-acceptor fluorophores (H2B-eGFP and H2B-mCh) is modulated by the underlying local nucleosome proximity on a scale of 1–10 nm (43). Here, to detect and spatially map this super-resolved readout of chromatin structure throughout HeLa, HeLa^KD^ and HeLa^HP1αI165E+KD^ co-expressing H2B-eGFP and H2B-mCh, we employed the phasor approach to FLIM, and in each diffraction limited pixel, measured the fluorescence lifetime of H2B-eGFP (donor), since this property is increasingly quenched upon closer FRET interaction with H2B-mCh (acceptor) (44,45) (Figure 3B). This involved first characterising the unquenched (donor control) versus quenched (due to histone FRET) donor fluorescence lifetime by phasor analysis of FLIM data acquired in HeLa nuclei transiently transfected with H2B-eGFP in the absence and presence of H2B-mCh (Supplementary Figure S12a–e). Then, definition of phasor cursors that select for these two states (Figure 3C) and enable the fraction of open chromatin (0 % FRET, teal cursor) versus compact chromatin (15 % FRET, red cursor) to be quantified across HeLa, HeLa^KD^ and HeLa^HP1αI165E+KD^ nuclei co-expressing the histone FRET pair (Figure 3D).

From application of this phasor-based cursor analysis to detection of histone FRET throughout FLIM data acquired within HeLa (Figure 3A-B, left column) and HeLa^KD^ (Figure 3A-B, middle column), we found the presence of HP1α (monomers and dimers) to induce a chromatin network that exhibits significantly more histone FRET than when absent (Figure 3D), and thus HP1α is indeed involved in reducing global nucleosome spacing in a living cell. Intriguingly, however, when we applied the phasor histone FRET analysis to FLIM data acquired within HeLa^HP1αI165E+KD^ (Figure 3A-B, right column), we found the presence of only HP1α monomers to induce a chromatin network that exhibits significantly less histone FRET that when absent (Figure 3D), which is indicative of an increase in nucleosome spacing. Collectively, this histone FRET result (Figure 3A–D), alongside our MNase digestion experiment (Figure 2E), suggests HP1α monomers to impart a global nucleosome spacing throughout live cell chromatin that is reduced upon HP1α dimer formation. This histone FRET result was verified in the HeLa^HP1α-KO^ cell line (Supplementary Figure S12f–g), upon stable expression of untagged HP1α (HeLa^HP1α-KO+HP1α^) versus HP1α_I165E_ (HeLa^HP1α-KO+HP1αI165E^) (Supplementary Figure S4), and transient co-transfection with the histone FRET reporter.

Since our histone FLIM-FRET analysis found HP1α dimer formation to reduce nucleosome proximity (Figure 3A–D), and HP1α foci formation was observed in NB and HP1α FLIM-FRET data to be a dimer dependent sub-micron structure (Figure 1A, H), we next investigated the spatial relationship between histone FRET and HP1α localisation in HeLa. Specifically, to test whether HP1α foci exhibit a more compact chromatin structure on a nanoscale compared to the surrounding chromatin network, which is diffusely decorated with HP1α, we performed a masked histone-FRET analysis of FLIM data acquired in HeLa co-expressing H2B-eGFP and H2B-mCh that was based on IF against HP1α versus H3K9me3 modified chromatin (Figure 3E-F). Interestingly, when applied across multiple HeLa nuclei, this IF masked histone FRET analysis demonstrated that on a nanoscale, HP1α foci (i.e. high intensity HP1α IF) are not underpinned by a more compact chromatin structure than the surrounding HP1α decorated chromatin network (Figure 3G). This result alongside the fact that H3K9me3 modified chromatin is associated with significantly more histone FRET (Figure 3H and Supplementary Figure S13), suggests that although the compaction status of chromatin marked for HP1α decoration is significantly higher than surrounding chromatin, its assembly into foci by HP1α dimers, does not further reduce nucleosome proximity. Instead, HP1α foci appear to be the result of more HP1α dimer bridged nucleosomes and chromatin fibres occupying these sub-micron spaces, alongside HP1α monomers that impart a global nucleosome spacing throughout live cell chromatin.

### HP1α monomers bind chromatin, and this interaction depends on H3K9me3 recognition

Histone FRET and MNase digestion suggest HP1α monomers to impart a nucleosome spacing throughout the chromatin network that is reduced upon HP1α dimer formation (Figure 2-3). This interpretation, however, requires confirmation that HP1α monomers: (i) have the capacity to bind chromatin, and (ii) in the absence of HP1α dimer formation, they do not interfere with the function of other HP1 isoforms (i.e. HP1β or HP1γ) or proteins involved in heterochromatin maintenance (e.g. SUV39H1). Thus, next, to investigate the binding affinity of HP1α monomers for chromatin, and their potential to undergo heterotypic interaction, we employed the phasor approach to FLIM for detection of HP1α FRET with histone H2B, alongside a series of one and two-channel FFS-based experiments, where the fluctuations recorded were analysed by a method called raster image correlation spectroscopy (RICS) (65). This required transient transfection of HeLa^eGFP-HP1α+KD^ and HeLa^eGFP-HP1αI165E+KD^ with either H2B-mCh for FRET analysis of HP1α chromatin binding, or a HP1 isotope / heterochromatin factor labelled with mCherry or a red fluorescent protein (RFP657) for cross RICS analysis of heterotypic interaction (66).

**Figure 4.**
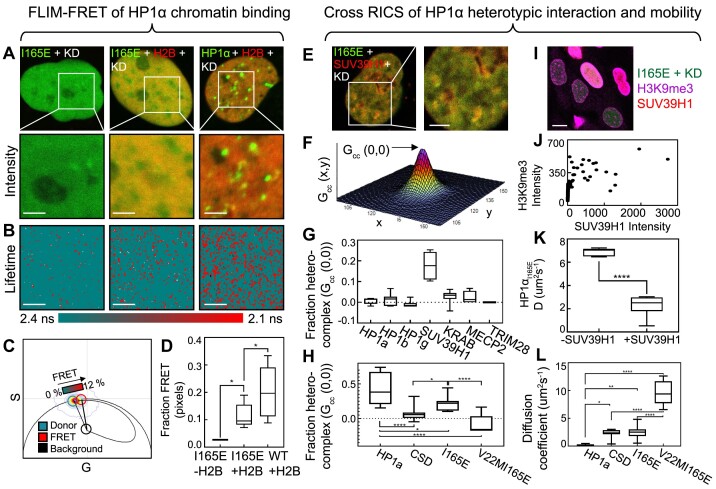
HP1α monomers bind chromatin via the SUV39H1 mediated H3K9me3 histone modification. (**A**) Merged intensity images of eGFP-HP1α_I165E_ in the absence (A, left) versus presence (A, middle) (FRET experiment) of H2B-mCh in HeLa^eGFP-HP1αI165E+KD^nuclei and eGFP-HP1α in the presence of H2B-mCh in a HeLa^eGFP-HP1α+KD^ nucleus (FRET experiment) (A, right) (top row) with in each case a selected region of interest (ROI) for FLIM data acquisition (A, bottom row). Scale bars 2 μm. (**B**-**C**) Lifetime maps of eGFP-HP1α_I165E_ versus eGFP-HP1α throughout the FLIM data acquisitions selected in (A) pseudo-coloured according to the palette defined in the phasor plot in (C) spatially map chromatin binding interaction (red pixels). Scale bars 2 μm. The phasor distribution of eGFP-HP1α_I165E_ detected throughout the lifetime maps in (B) is superimposed with a theoretical FRET trajectory that enables the efficiency of FRET with H2B-mCh (red cursor) to be characterised, given our independent determination of the unquenched donor fluorescence lifetime (teal cursor) (right column in A-B). (**D**) Quantification of the fraction of pixels exhibiting FRET (i.e. chromatin binding) across multiple HeLa^eGFP-HP1αI165E+KD^ and HeLa^eGFP-HP1α+KD^ nuclei co-expressing H2B-mCh (N ≥ 7 cells, two biological replicates). (**E**) Merged intensity image of a HeLa^eGFP-HP1αI165E+KD^ nucleus co-expressing SUV39H1-mCh and a selected ROI for cross RICS data acquisition. Scale bar 2 μm. (**F**) Analysis of the cross RICS data acquisition in (E) results in a cross RICS profile that exhibits an amplitude (G_cc_(0,0)) indicative of the fraction of heterocomplex present in the selected ROI. (**G**) Quantification of the fraction of eGFP-HP1α_I165E_ in a hetero-complex with the potential binding partners (*N* = 9 cells, two biological replicates). (**H**) Quantification of the fraction of eGFP-HP1α mutants in a hetero-complex with the SUV39H1 (*N* ≥ 9 cells, two biological replicates). (**I**) Merged intensity images of eGFP-HP1α_I165E_ (green), H3K9me3 (magenta) and SUV39H1-mCh (red) in HeLa^HP1αI165E+KD^ cells transiently expressing SUV39H1-mCh. Scale bars 10 μm. (**J**) The intensity plot of SUV39H1-mCh versus H3K9me3 across multiple nuclei (*N* = 190 cells, one biological replicate). (**K**) The diffusion coefficient of eGFP-HP1α_I165E_ in the presence versus absence of SUV39H1-mCh expression (*N* ≥ 7 cells, two biological replicates). (**L**) The diffusion coefficient of eGFP-HP1α mutants in the presence of SUV39H1-mCh expression. The box and whisker plots in (D), (G-H) and (K-L) show the minimum, maximum, sample mean: *P< 0.05, ** *P*< 0.01, ****P< 0.0001, one-way ANOVA.

For phasor FLIM-FRET analysis of the HP1α monomer's capacity to bind chromatin we first characterised the unquenched fluorescence lifetime of eGFP-HP1α_I165E_ (donor control) by phasor analysis of FLIM data acquired in HeLa^eGFP-HP1αI165E+KD^ and then compared this with the FRET quenched fluorescence lifetime of: (i) eGFP-HP1α_I165E_ in HeLa^eGFP-HP1αI165E+KD^ transiently transfected with H2B-mCh (readout of HP1α monomer binding affinity), and (ii) eGFP-HP1α in HeLa^eGFP-HP1α+KD^ transiently transfected with H2B-mCh (readout of HP1α monomer and dimer binding affinity) (Figure 4A-B). Collectively, this FLIM data enabled definition of phasor cursors that select for unbound HP1α (i.e. unquenched eGFP with 0 % FRET) versus chromatin bound HP1α (i.e. quenched eGFP with 12 % FRET) (Figure 4C), and the fraction of HP1α monomer and or dimer interaction with chromatin to be quantified (Figure 4D) from the FLIM maps (Figure 4B). From application of this phasor-based cursor analysis to multiple cells (Figure 4D), we found that a significant fraction of HP1α monomers do indeed bind chromatin (11 ± 2 %), albeit with less affinity in the absence of HP1α dimer formation (20 ± 3 %); a result orthogonally confirmed via a comparative RICS analysis of eGFP-HP1α_I165E_ mobility that is a proxy for chromatin binding capacity (Supplementary Figure S14). Thus, we conclude that monomeric HP1α does have the capacity to bind chromatin and directly impart the spacing between nucleosomes that was detected by MNase digestion (Figure 2) and FLIM analysis of histone H2B FRET (Figure 3).

To next interrogate the HP1α monomer's potential for heterotypic interaction by cross RICS analysis we acquired a series of two-channel FFS datasets in HeLa^eGFP-HP1αI165E+KD^ transiently transfected with one of the following potential HP1α monomer binding partners: HP1α-mCh, HP1β-mCh, HP1γ-mCh, SUV39H1-mCh, KRAB-RFP657, MeCP2-RFP657 and RFP657-Trim28 (Figure 4E). Then, for each two-channel FFS dataset, we spatially cross correlated the spectrally distinct fluorescence fluctuations recorded in the eGFP-HP1α_I165E_ and mCherry (or RFP657) channels by application of the cross RICS function (Supplementary Figure S15a–c) and employed the amplitude of a diffusion model fit to each resulting cross RICS profile as a readout of the fraction of heterocomplex detected (Figure 4F). From quantification of the cross RICS amplitude across multiple nuclei we found that while eGFP-HP1α_I165E_ does not interact with HP1β or HP1γ (Figure 4G); nor compete with these HP1 isoforms’ capacity to interact with chromatin, according to a series of FLIM-FRET experiments with H2B (Supplementary Figure S16); it is incorporated into a heterocomplex with histone-lysine *N*-methyltransferase (SUV39H1) (Figure 4G); the enzyme responsible for deposition of H3K9me3; in a manner that depends on recognition of H3K9me3 according to a series of cross RICS experiments with mutants of HP1α (Figure 4H) and does not disrupt SUV39H1 function according to quantitative intensity imaging of H3K9me3 (Supplementary Figure S17).

Since monomeric HP1α is dependent on H3K9me3 recognition for incorporation into a complex with SUV39H1 (Figure 4H), we next decided to investigate whether monomeric HP1α is also dependent on H3K9me3 for binding to chromatin. To do so we employed the fact that overexpression of SUV39H1-mCh leads to an increase in the H3K9me3 histone modification (Figure 4I, J), acquired a series of single-channel FFS experiments in HeLa^eGFP-HP1αI165E+KD^ nuclei that were negative versus positive for a transient transfection with SUV39H1-mCh, and spatially correlated the fluorescence fluctuations recorded throughout the eGFP-HP1α_I165E_ channel by application of the RICS function. Then from fitting the decay of each RICS function to a diffusion model and comparing the impact of SUV39H1-mCh on the recovered diffusion coefficient of eGFP-HP1α_I165E_ that we again use as a proxy for chromatin binding capacity, we found SUV39H1 induced H3K9me3 deposition to significantly reduce HP1α_I165E_ mobility (Figure 4K) in a manner that depends on HP1α_I165E_ recognition of this epigenetic mark (Figure 4L). Thus, this result alongside eGFP-HP1α_I165E_-H2B-mCh FLIM-FRET (Figure 4D) suggests that HP1α monomer chromatin binding is promoted by nucleosomes that present H3K9me3.

## Discussion

The mechanism by which HP1α dimers fold H3K9me3 modified nucleosomes into a higher order chromatin network structure that silences gene expression, has not been elucidated in a living cell, because nucleosome proximity is not resolvable by standard optical microscopy. Thus, here to address this knowledge gap, we coupled molecular editing in HeLa^HP1α-KD^ and HeLa^HP1α-KO^ cell systems with a multiplexed approach to advanced fluorescence microscopy, which enabled direct observation of the impact HP1α dimer formation has on nuclear wide chromatin organisation down to the nanoscale. In doing so, we were able to demonstrate that under physiological conditions, approximately 80 % of HP1α molecules exist as a monomer throughout the nucleus of a living cell and these HP1α monomers upon binding H3K9me3 decorated chromatin, impart a nuclear wide spacing between nucleosomes that can be locally reduced upon HP1α dimer formation. The net effect of these antagonistic HP1α functions is a sub-micron heterochromatin architecture that is actively moderating collapse of DNA into nuclear condensates and regulated by chromatin compaction on a nanoscale.

**Figure 5.**
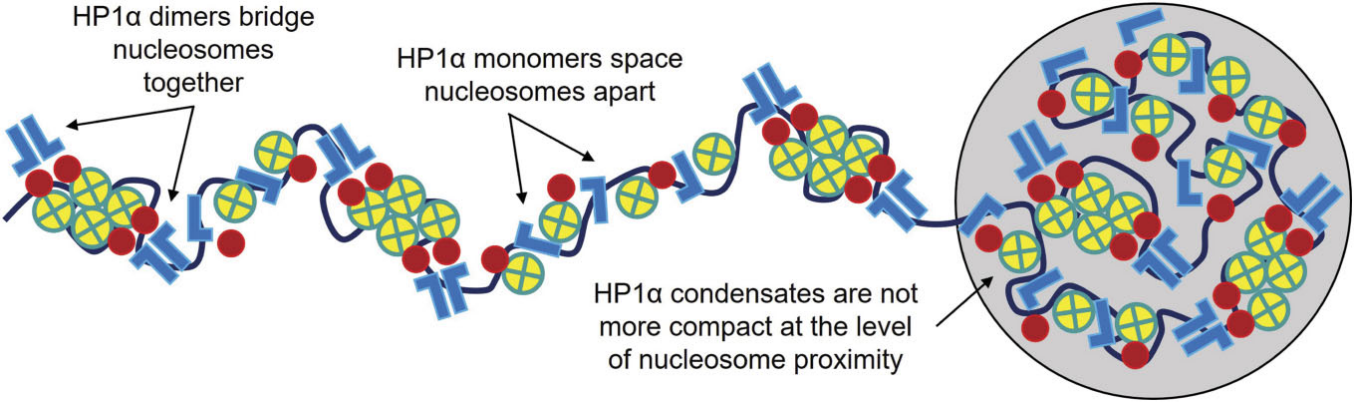
Schematic of the HP1α monomer to dimer transition modulating nucleosome proximity. HP1α monomers and dimers (blue) upon binding H3K9me3 modified (red) nucleosomes (yellow), space apart and bridge together nucleosomes to locally regulate DNA template access. This nanoscale organisation in nucleosome proximity is unchanged by HP1α nuclear condensate formation.

In terms of novelty, the first aspect of our findings on heterochromatin architecture that was particularly new, was the fact that the HP1α monomer appears to serve an active role in maintenance of chromatin network architecture that goes beyond simply serving as a precursor to the HP1α dimer. Specifically, while *in vitro* studies have demonstrated HP1α monomers exhibit a binding affinity for chromatin that is significantly increased upon HP1α dimer formation (10,35,36,75), to our knowledge we are the first to detect a nucleosome spacing event when HP1α monomers bind chromatin, which precedes HP1α dimer bridging of nucleosomes and thus increases the dynamic range of nucleosome proximities that are possible between open versus compact chromatin when regulating access to the DNA template (Figure 5, left). This result potentially offers a mechanistic explanation for previous studies that have found HP1α in certain contexts to maintain chromatin open despite its well-established role in compaction (42,76). It is also important to note that the detected antagonistic function of the HP1α monomer could be mediated by its incorporation into a heterotypic complex with other factors (77–79).

A second aspect of our findings on heterochromatin architecture that is important to note, is the fact that HP1α foci, when compared to the surrounding chromatin network marked by H3K9me3 for HP1α decoration, are not underpinned by a more compact chromatin structure on a nanoscale. Instead, HP1α foci, which co-localise with DNA condensates that can form in the absence of HP1α expression, are underpinned by a higher concentration of HP1α dimer bridged and monomer spaced nucleosomes within these sub-micron volumes (17), rather than a more closely packed organisation of these nanometre sized sub-units (Figure 5, right). This result highlights the fact that diffraction limited visualisation of dense nuclear structures like HP1α foci is not necessarily indicative of where chromatin nanostructure is compact (42,43,80), and supports findings obtained by electron microscopy of chromatin (chromEMT) that demonstrate 3D domains inside the nucleus are the result of different chromatin concentrations rather than discrete higher order chromatin structures (18).

Thus, in conclusion, our results demonstrate that sub-micron heterochromatin architecture is maintained by a HP1α monomer to dimer transition that differentially regulates nucleosome proximity, and we detected this result by modulating the capacity of HP1α to self-associate and microscopy-based measurement of the impact this molecular perturbation had on chromatin structure down to a nanoscale. What remains unclear, however, is where or how the chromatin bound HP1α monomers impart a spacer between nucleosomes that can be locally reduced at any time or chromatin location by dimer HP1α formation, and the physiological role of HP1α monomer spacing in, for example, gene expression or maintenance of genome integrity? Thus, in future studies it would be interesting to explore *in vitro* and *in silico* whether HP1α monomers open chromatin by volume exclusion and or reshaping the nucleosome core (14), comprehensively survey whether HP1α monomers open chromatin via incorporation into a heterocomplex (77–79), investigate the impact of these nanoscale events on nuclear mechanics during cell division (81), and design experiments that can further investigate the impact of HP1α monomers versus dimers on transcription and or DNA repair dynamics in a living cell (82).

## Supplementary Material

gkae720_Supplemental_File

## Data Availability

The datasets generated during and/or analysed during the current study are available from the corresponding author on reasonable request.
