# Supplementary material for: Heterochromatin protein 1 alpha (HP1α) undergoes a monomer to dimer transition that opens and compacts live cell genome architecture
Source: Nucleic Acids Res. 2024 Aug 28;52(18):10918–33. doi: 10.1093/nar/gkae720 (PMC11472067; doi:10.1093/nar/gkae720)
Supplement: gkae720_Supplemental_File [file gkae720_supplemental_file.pdf]

Supplementary Information

Heterochromatin protein 1 alpha (HP1α) undergoes a monomer to dimer transition that opens and compacts live cell genome architecture.

Jieqiong Lou[1], Qiji Deng[2], Xiaomeng Zhang[1], Charles C. Bell[2], Andrew B. Das[2,3], Naiara G. Bediaga[2], Courtney O. Zlatic[4], Timothy M. Johanson[5,6], Rhys S. Allan[5,6], Michael D. W. Griffin[4], Prasad. N. Paradkar[7], Kieran F. Harvey[2,3,8], Mark A. Dawson[2,3,9], Elizabeth Hinde[1,4*]

1. School of Physics, University of Melbourne, Melbourne, VIC 3010, Australia.
2. Peter MacCallum Cancer Centre, 305 Grattan St, Melbourne, VIC 3000, Australia.
3. Sir Peter MacCallum Department of Oncology, University of Melbourne, Parkville, VIC 3010, Australia.
4. Department of Biochemistry and Pharmacology, Bio21 Molecular Science and Biotechnology Institute, University of Melbourne, Melbourne, VIC 3010, Australia.
5. The Walter and Eliza Hall Institute of Medical Research, Parkville, VIC 3052, Australia
6. Department of Medical Biology, The University of Melbourne, Parkville, VIC 3010, Australia.
7. CSIRO Health & Biosecurity, Australian Centre for Disease Preparedness, 5 Portarlington Road, Geelong 3220, Australia.
8. Department of Anatomy and Developmental Biology and Biomedicine Discovery Institute, Monash University, Clayton, VIC 3168, Australia.
9. Centre for Cancer Research, University of Melbourne, Melbourne, VIC 3010, Australia.

*Corresponding author: elizabeth.hinde@unimelb.edu.au

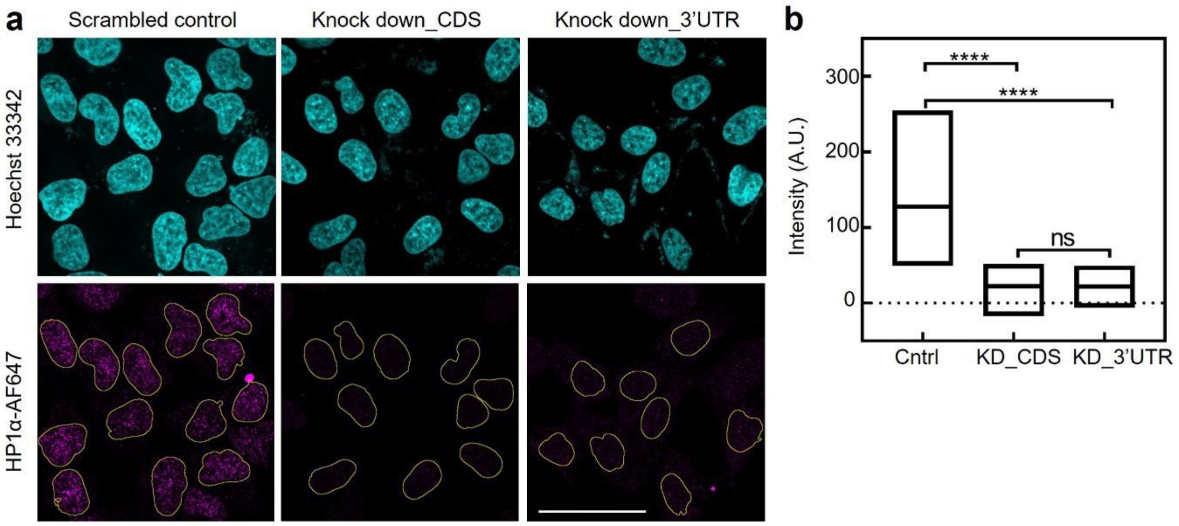

Supplementary Figure 1. Knock down (KD) of endogenous HP1α via use of siRNA targeting HP1α's mRNA coding sequence (CDS) versus 3' untranslated region (3'UTR). (**a**) Representative intensity images of DNA (Hoechst 33342) versus HP1α immunofluorescence (IF) (HP1α-AF647) in HeLa cells treated with scrambled siRNA (left) versus siRNA targeting of HP1α's mRNA coding sequence (CDS) (middle) and 3'UTR (right). Scale bar 50 μm. (**b**) Quantification of HP1α expression under endogenous versus an siRNA knock down condition across multiple cells (N > 115 cells, two biological replicates). The floating bar plot in (b) shows the minimum, maximum, sample median: **** $P < 0.0001$, ns $P > 0.05$, one-way ANOVA.

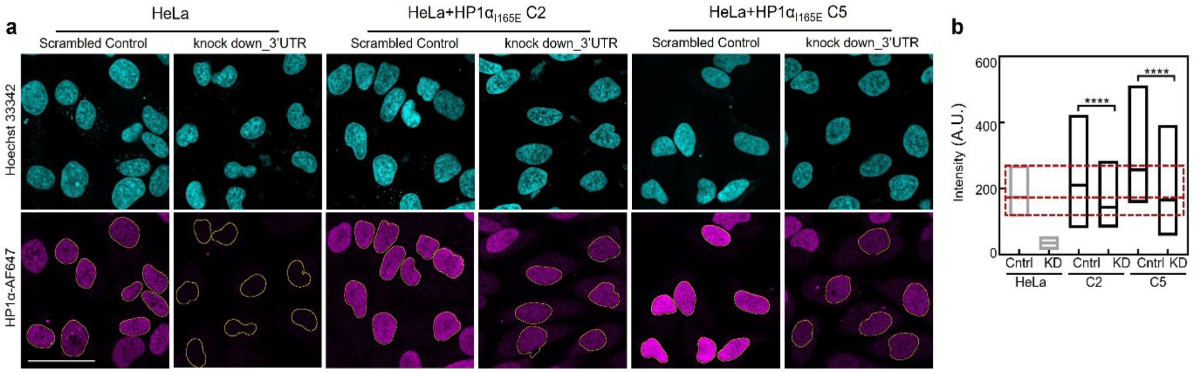

Supplementary Figure 2. Establishing untagged HP1α$_{I165E}$ stable expression in HeLa cells. (a) Representative DNA (top row) and HP1α IF intensity images (bottom row) in HeLa, HeLa$^{HP1\alpha I165E}$ clone 2 (C2), and HeLa$^{HP1\alpha I165E}$ clone 5 (C5) cell lines treated with scrambled siRNA or siRNA targeting HP1α 3'UTR. Scale bar 50 µm. **(b)** Quantification of HP1α expression under the endogenous versus knock down (KD) conditions in the HeLa, C2, and C5 cell lines across multiple cells; red square indicates the IF intensity range of endogenous HP1α in HeLa cells (N > 91 cells, two biological replicates). The floating bar plot in (b) shows the minimum, maximum, sample median: **** $P < 0.0001$, unpaired t-test.

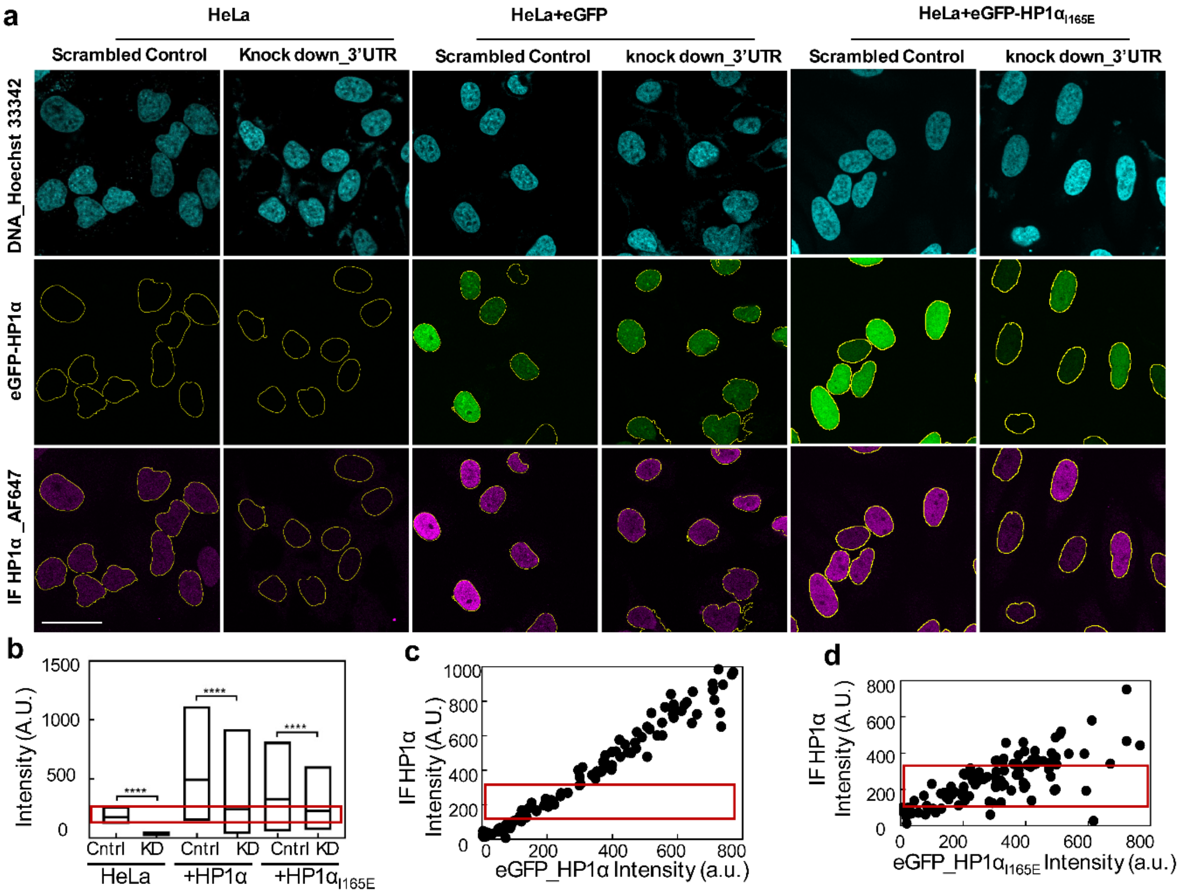

Supplementary Figure 3. IF against endogenous HP1α in HeLa enables identification of eGFP intensity in HeLa$^{eGFP\text{-}HP1\alpha}$ and HeLa$^{eGFP\text{-}HP1\alpha I165E}$ that correlates with a physiological expression level. (a) Representative intensity images of DNA (Hoechst 33342) (top row), eGFP intensity (middle row), and HP1α immunofluorescence (IF) (HP1α-AF647) in HeLa, HeLa$^{eGFP\text{-}HP1\alpha}$, and HeLa$^{eGFP\text{-}HP1\alpha I165E}$ treated with scrambled siRNA or siRNA targeting HP1α 3'UTR. HeLa$^{eGFP\text{-}HP1\alpha}$ and HeLa$^{eGFP\text{-}HP1\alpha I165E}$ were also treated with 100 µg / ml of cycloheximide for 3 h before fixation. Scale bar 50 µm. **(b)** Quantification of HP1α expression under the endogenous versus knock down (KD) conditions in HeLa, HeLa$^{eGFP\text{-}HP1\alpha}$, and HeLa$^{eGFP\text{-}HP1\alpha I165E}$ cell lines across multiple cells; red square indicates the IF intensity range of endogenous HP1α in HeLa cells (N > 50 cells, two biological replicates). **(c-d)** Plots of eGFP-HP1α (c) and eGFP-HP1α$_{I165E}$ (d) versus HP1α IF intensity in HeLa$^{eGFP\text{-}HP1\alpha}$ (c) and HeLa$^{eGFPHP1\alpha I165E}$ cells (d) treated with siRNA targeting HP1α 3'UTR (i.e., HeLa$^{eGFP\text{-}HP1\alpha\text{+}KD}$ and HeLa$^{eGFP\text{-}HP1\alpha I165E\text{+}KD}$): red square indicates the IF intensity range of endogenous HP1α in HeLa cells as determined in panel (b). HeLa$^{eGFP\text{-}HP1\alpha\text{+}KD}$ and HeLa$^{eGFP\text{-}HP1\alpha I165E\text{+}KD}$ cells exhibiting a mean eGFP-HP1α and eGPP-HP1α$_{I165E}$ intensity within the red square presented in (b-d) were selected for all imaging experiments investigating HP1α protein interaction dynamics. The floating box plots in (b) show the minimum, maximum, sample median: **** $P < 0.0001$, unpaired t-test.

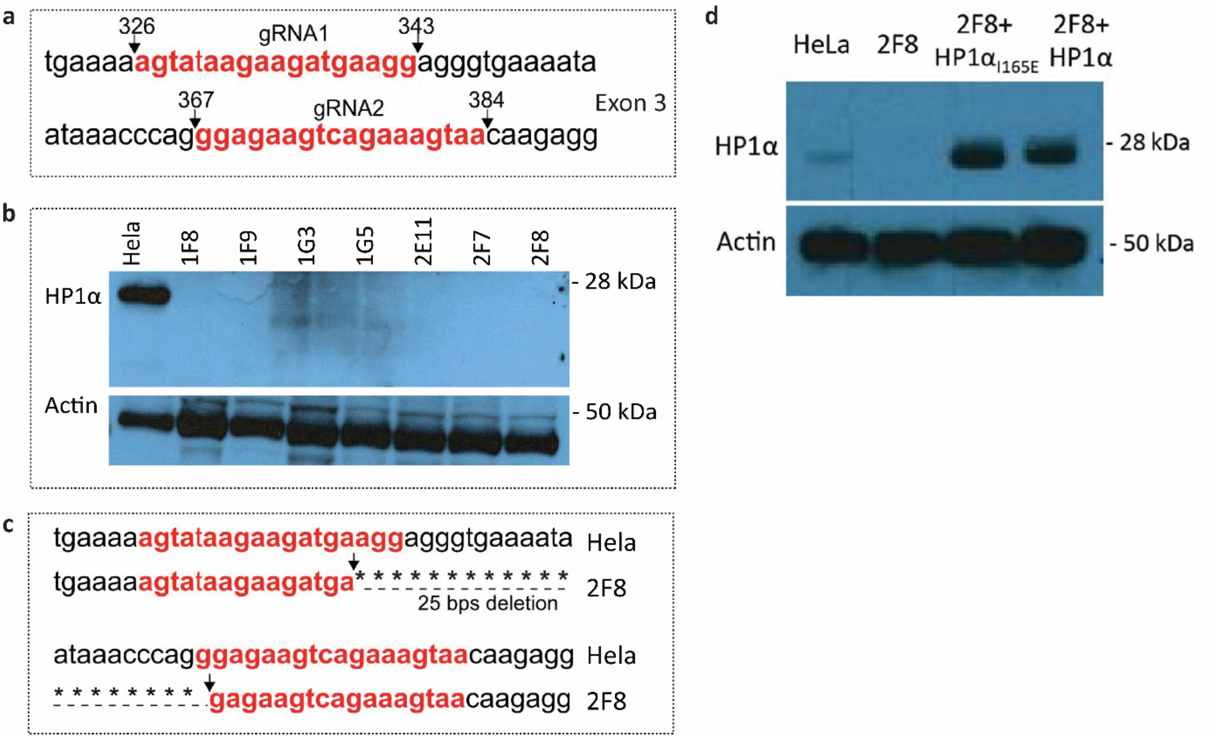

Supplementary Figure 4. Knock out (KO) of HP1α (HeLa$^{HP1\alpha\ KO}$) in HeLa by CRISPR/Cas9 and the stable expression of HP1α (HeLa$^{HP1\alpha\ KO+HP1\alpha}$) versus HP1α$_{I165E}$ (HeLa$^{HP1\alpha\ KO+HP1\alpha I165E}$). (a) Two gRNAs targeting human HP1α exon 3 were designed. **(b)** Western blot demonstrated that multiple single cell clones, including clone 2F8 had no HP1α protein detected. **(c)** Genomic DNA sequence demonstrated that clone 2F8 had a 25 bp nucleotide deletion in between gRNA1 and gRNA2. **(d)** Western blot detecting HP1α in the HeLa, HeLa$^{HP1\alpha\ KO}$, HeLa$^{HP1\alpha\ KO+HP1\alpha I165E}$, and HeLa$^{HP1\alpha\ KO+HP1\alpha}$ cell lines.

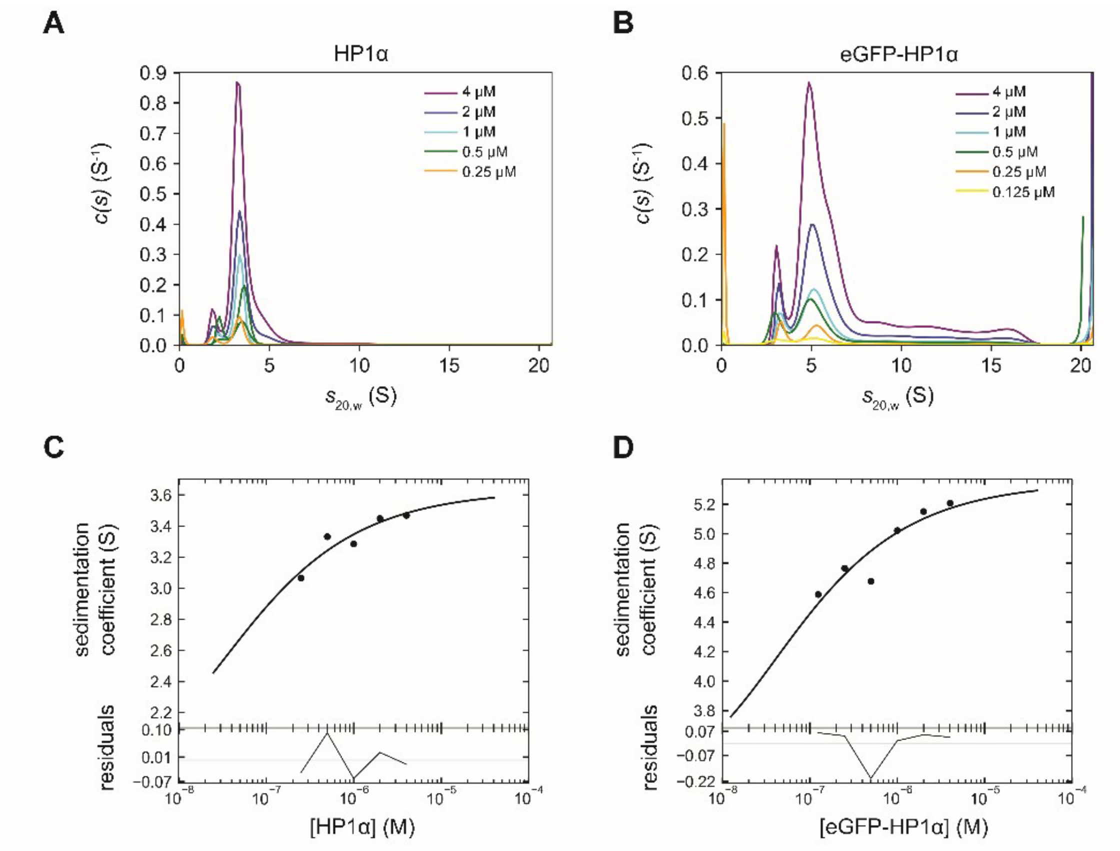

Supplementary Figure 5. Sedimentation velocity analytical ultracentrifugation (SV-AUC) reveals the HP1α dimer dissociation constant (KD) to be unaltered by an eGFP tag. (a) Continuous sedimentation coefficient ($c(s_{20,w})$) distributions for HP1α at concentrations from 0.25 µM to 4 µM. (**b)** Continuous sedimentation coefficient (c(s20,w)) distributions for eGFPHP1α at concentrations from 0.125 µM to 4 µM. (**c)** Fit of the weight-average sedimentation coefficient isotherm derived from data shown in (a) (black circles) to a monomer-dimer selfassociation model (solid line). The calculated K_D was 57 nM (68% confidence interval 19 - 121 nM) consistent with upper bound estimates by Her et al. (doi:10.1093/nar/gkac1194). (**d)** Fit of the weight-average sedimentation coefficient isotherm derived from data shown in (b) (black circles) to a monomer-dimer self-association model (solid line). The calculated K_D was 68 nM (68% confidence interval 25 - 137 nM). The lowest concentration measured for each sample was determined by the dynamic range of the Optima AUC UV-visible detection system. The higher extinction coefficient of eGFP-HP1α allowed measurement of a lower concentration of this construct, relative to untagged HP1α, and this measurement was incorporated into the analysis (b) and (d). The sedimentation coefficient of the dimer was fit for each isotherm. Due to limited fitting restraints at lower concentrations, the monomer sedimentation coefficients were fixed at 1.8 S and 3.3 S for HP1α (c) and eGFP-HP1α (d), based on the lower bounds of the c($s_{20,w}$) distributions in (a) and (b). Signal above 8 S was shown to be non-interacting and was excluded from analysis. It should also be noted that while the calculated K_D for HP1α versus eGFP-HP1α is informative in demonstrating that the eGFP tag does not substantially impact the propensity for HP1α to self-associate, these values do not necessarily predict the fraction of HP1α versus eGFP-HP1α that dimerises and oligomerises in a living cell at any given concentration because a test tube omits the complexity of the nuclear environment (Yang et al. 2020 PNAS, Rivas et al. 2018 Biophys Rev) and competing heterotypic protein interactions (Komatsubara et al. 2019 JBC, Sadaie et al. 2014 MCB).

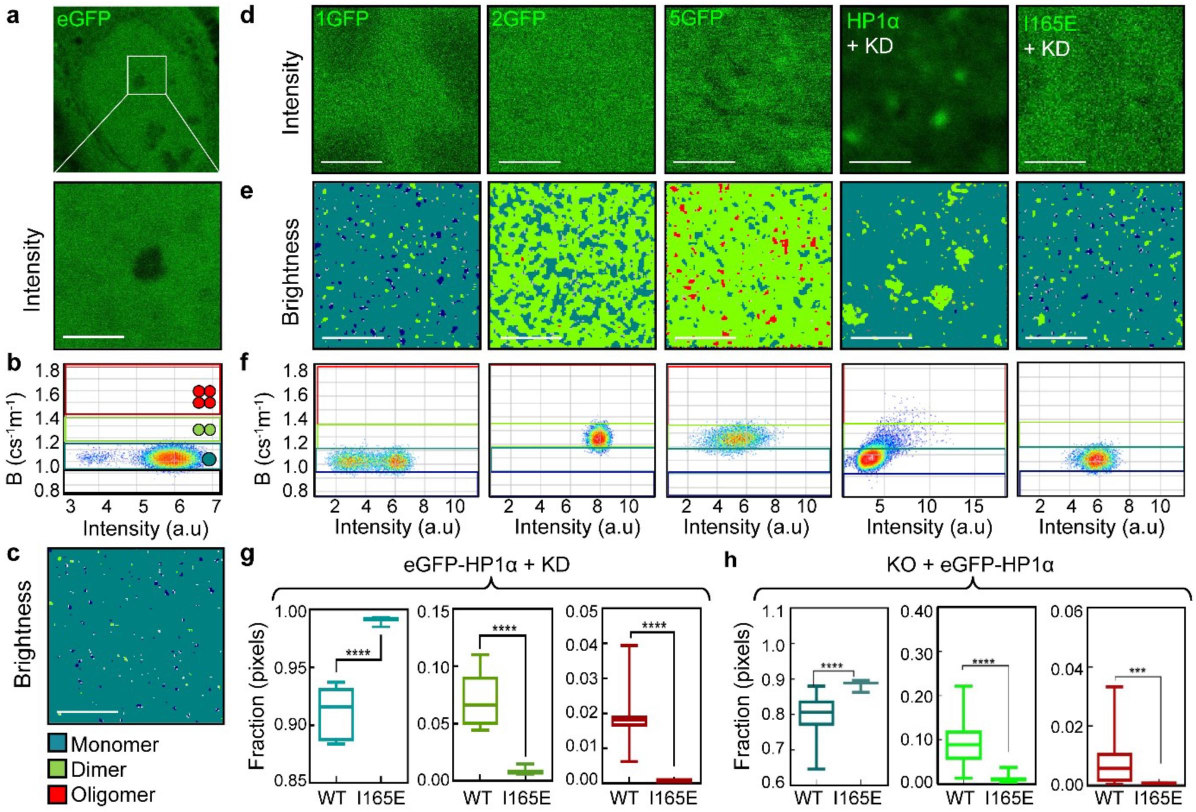

Supplementary Figure 6. Number and Brightness (NB) analysis of HP1α oligomerisation in a living cell. (a) Intensity image of the eGFP signal throughout a selected HeLa cell (a. top) and the region of interest (ROI) from which a NB data acquisition was recorded (a. bottom). Scale bars 2 µm. (**b)** Intensity versus brightness scatterplot of the eGFP NB data acquisition presented in (a) with brightness windows extrapolated from this monomeric calibration (teal cursor) for detection of eGFP dimers (light green cursor) and oligomers (red cursor) **(c)** Brightness map of the NB data acquisition presented in (a) pseudo-coloured according to the brightness windows defined in (b) spatially maps the HP1α monomer brightness distribution (teal). (**d-f)** Validation of the eGFP monomer extrapolated brightness windows defined in (b) on GFP multimers (1GFP, 2GFP and 5GFP) and their application to HP1α self-association versus inhibition (HP1α and I165E). Note that in the case of the 5GFP construct it has a propensity to degrade into smaller subunits based on Western Blot analysis and so upon expression in live cells we detect a heterogenous combination of dimers and oligomers. (**g-h)** Comparison of NB quantification of HP1α self-association versus inhibition (HP1α and I165E) in HeLa$^{eGFP\text{-}HP1\alpha+KD}$ and HeLa$^{eGFP\text{-}HP1\alpha I165E+KD}$ (i.e., knock down (KD) conditions) versus in HeLa$^{KO+eGFP\text{-}HP1\alpha}$ HeLa$^{KO+eGFP\text{-}HP1\alpha I165E}$ (i.e., knock out (KO) conditions). The box and whisker plots in (g-h) show the minimum, maximum, sample mean: $**P<0.01$, $****P<0.0001$, unpaired *t*-test.

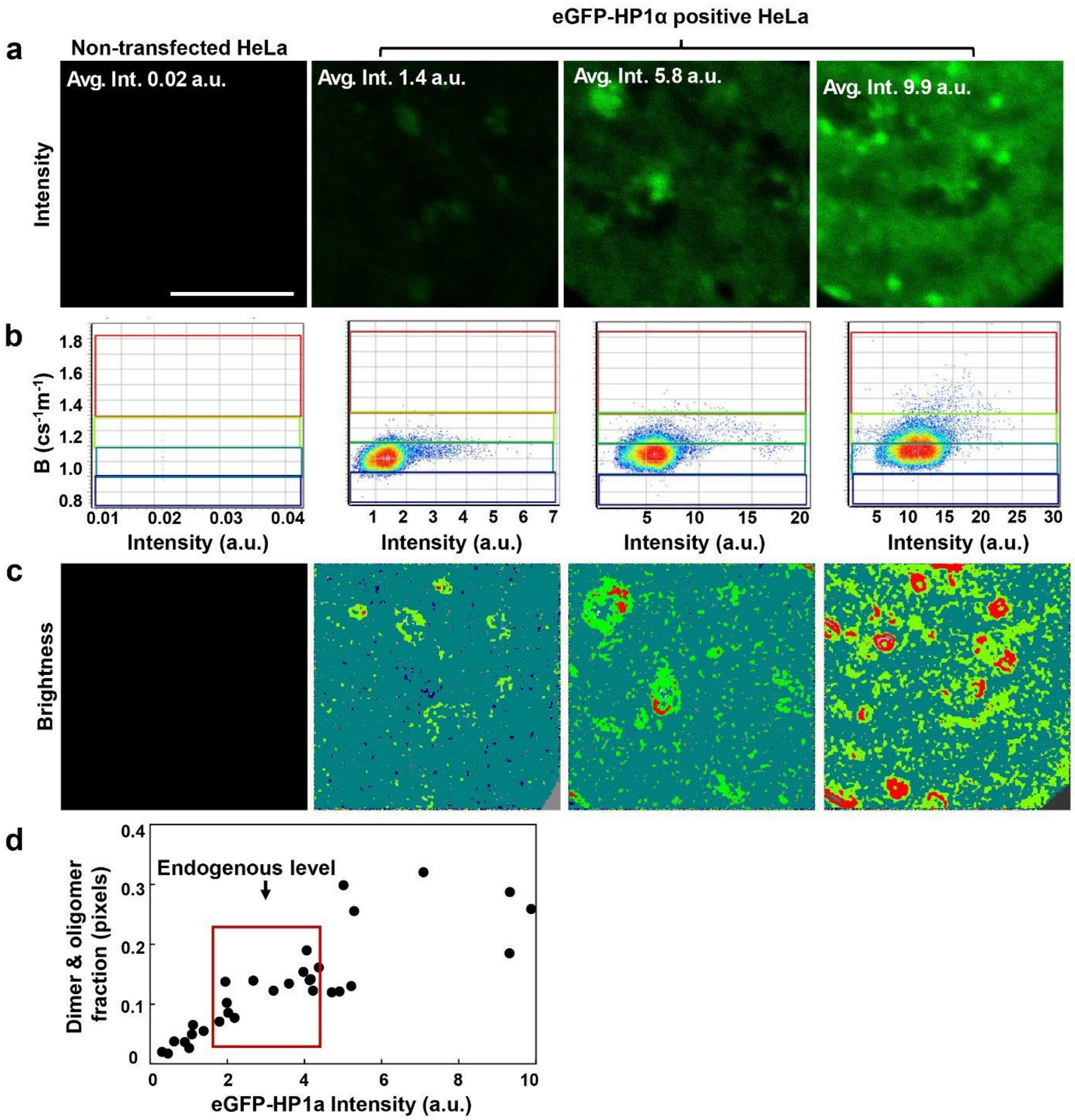

Supplementary Figure 7. NB analysis of HP1α oligomerisation in HeLa cells exhibiting different eGFP-HP1α expression levels. (a) Intensity image of the eGFP-HP1α signal throughout selected HeLa cells exhibiting different eGFP-HP1α expression levels. Scale bars 2 μm. **(b)** Intensity versus brightness scatterplots of the NB data acquisitions presented in (a) with brightness windows extrapolated from the monomeric eGFP calibration (teal cursor) for detection of eGFP-HP1α dimers (green cursor) and oligomers (red cursor). **(c)** Brightness maps of the NB data acquisitions presented in (a) pseudo-coloured according to the brightness windows defined in (b) spatially map HP1α monomer (teal), dimer (green) and oligomer (red) localisation. **(d)** A plot of the impact of eGFP-HP1α expression level (i.e., intensity) on the detected fraction of eGFP-HP1α dimer and oligomer formation in HeLa cells: red square indicates cells with an endogenous HP1α expression level.

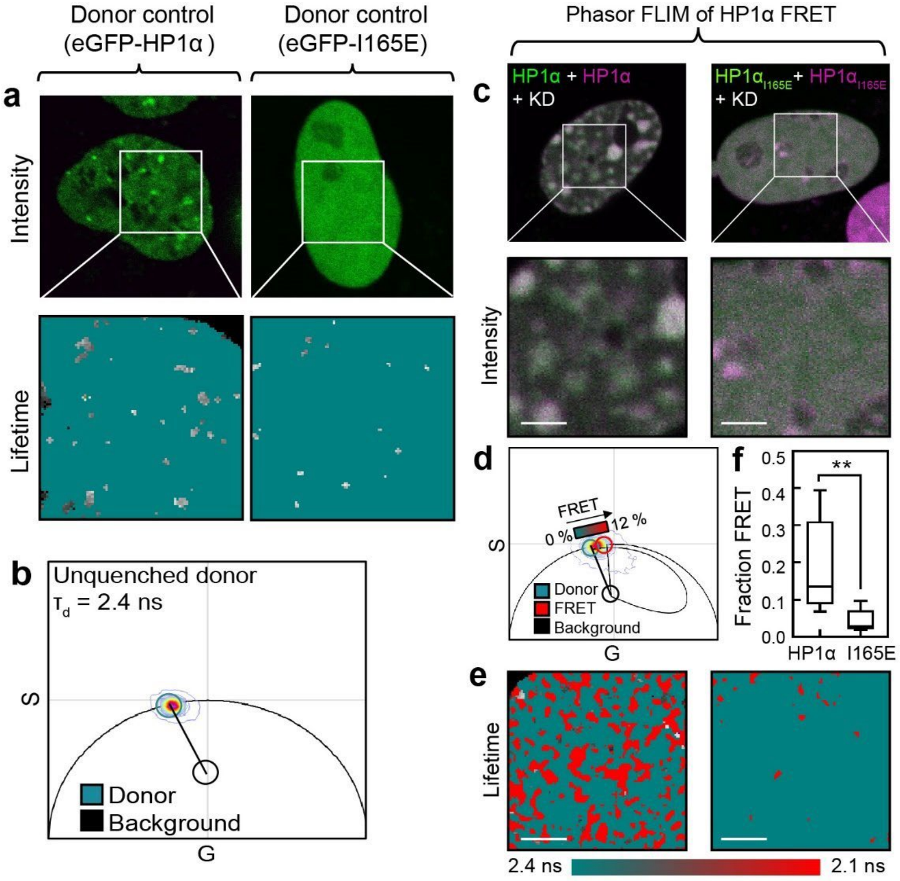

Supplementary Figure 8. Fluorescence lifetime imaging microscopy (FLIM) of HP1α hetero FRET as an orthogonal readout of HP1α oligomerisation in a living cell. (a-b) Intensity images of a HeLa$^{eGFP\text{-}HP1\alpha+KD}$ versus HeLa$^{eGFP\text{-}HP1\alpha I165E+KD}$ nucleus (top) with a lifetime map derived from a FLIM data acquisition within a selection region of interest (ROI) (bottom) (a) that is pseudo-coloured according to the palette defined in the phasor plot (b). This represents a donor control that enables characterisation of the unquenched fluorescence lifetime of eGFP-HP1α and eGFP-HP1α$_{I165E}$ as 2.4 ns. **(c)** Merged intensity images of a HeLa$^{eGFPHP1\alpha+KD}$ nucleus expressing RFP657-HP1α and a HeLa$^{eGFP\text{-}HP1\alpha I165E+KD}$ nucleus expressing RFP657-HP1α$_{I165E}$ (top) alongside the ROI from which each FLIM data acquisition was recorded (bottom). Scale bars 2 µm. **(d)** Phasor distribution of eGFP-HP1α fluorescence lifetime from panel (c) with a theoretical FRET trajectory superimposed to determine the efficiency of eGFP-HP1α FRET with RFP657-HP1α. The linear combination of unquenched donor and background cellular autofluorescence (teal-black) follows a distinct trajectory from FRET (teal-red) (panel b). **(e)** Lifetime maps of the FLIM data acquisitions presented in (c) pseudo-coloured according to the palette defined in the phasor plot in (d) spatially map HP1α monomers (teal) versus dimers and oligomers (red). **(f)** Quantification of the fraction of pixels exhibiting FRET (our read out of self-association) across multiple HeLa$^{eGFP\text{-}HP1\alpha+KD}$ versus HeLa$^{eGFP\text{-}HP1\alpha I165E+KD}$ nuclei transiently transfected with RFP657-HP1α or RFP657-HP1α$_{I165E}$ ($N \geq 8$ cells, one biological replicate). The box and whisker plot in (f) shows the minimum, maximum, sample mean: $^{**}P < 0.01$, unpaired t-test.

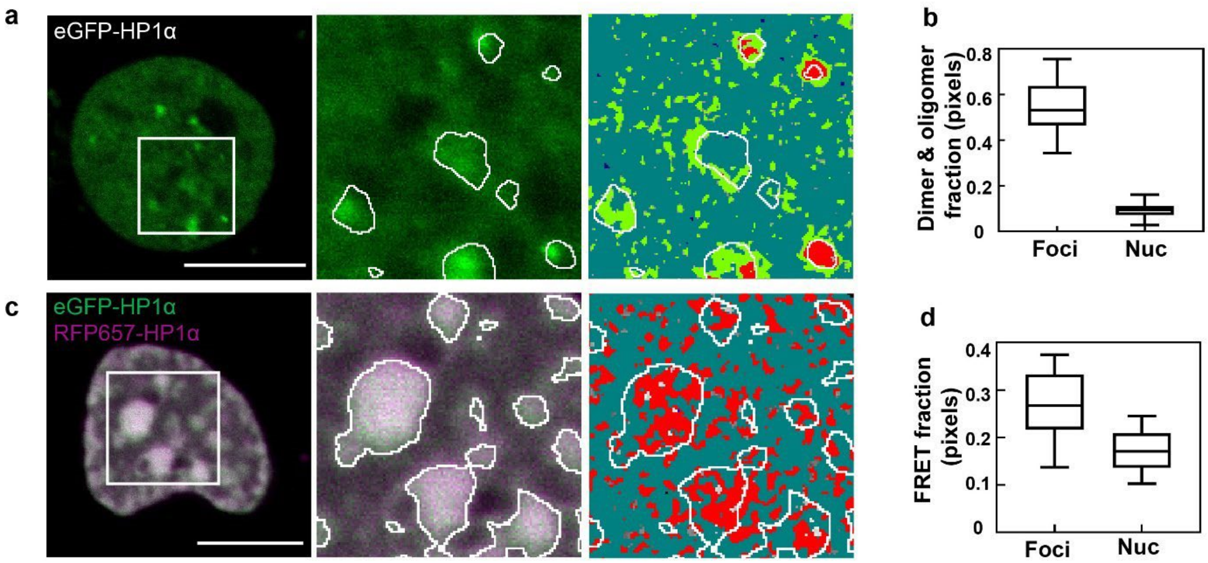

Supplementary Figure 9. Quantification of HP1α self-association in heterochromatin foci versus nucleoplasm. (**a**) Intensity image of a HeLa$^{eGFP\text{-}HP1\alpha+KD}$ nucleus (left) alongside the region of interest (ROI) from which the NB data acquisition was recorded (middle) and the corresponding brightness map pseudo-coloured according to the brightness windows defined in Supplementary Figure 6, spatially map HP1α monomer (teal), dimer (green) and oligomer (red) localisation. HP1α enriched heterochromatin foci (highlighted by white lines) were identified based on an eGFP-HP1α intensity threshold. Scale bars 10 µm. **(b)** Quantification of the HP1α dimer and oligomer fraction in heterochromatin foci versus nucleoplasm across multiple nuclei (N= 14 cells, two biological replicates) that is presented in Figure 1I. (**c**) Merged intensity image of a HeLa$^{eGFP\text{-}HP1\alpha+KD}$ nucleus expressing RFP657-HP1α (left) alongside the ROI from which the FLIM data acquisition was recorded (middle) and the corresponding lifetime map that pseudo-coloured according to the palette defined in the phasor plot in Supplementary Figure 8, spatially map HP1α monomers (teal) versus dimers and oligomers (red). HP1α enriched heterochromatin foci (highlighted by white lines) were identified based on the eGFP-HP1α intensity threshold. Scale bars 10 µm. **(d)** Quantification of the HP1α dimer and oligomer fraction in heterochromatin foci versus nucleoplasm across multiple nuclei (N= 8 cells, 1 biological replicate).

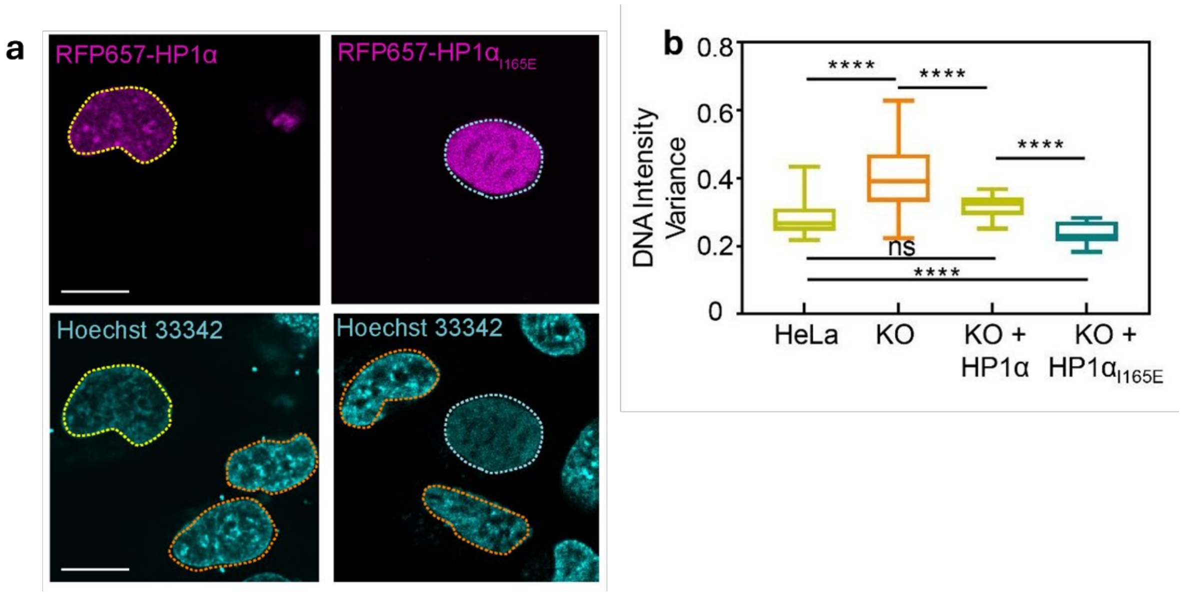

Supplementary Figure 10. CV analysis of chromatin density throughout Hela$^{HP1α\text{-}KO}$ transiently expressing RFP657-HP1α versus RFP657-HP1α$_{I165E}$. (a) Representative intensity images of Hoechst 33342 stained DNA density (blue) throughout the nuclei of Hela$^{HP1α\text{-}KO}$ transiently expressing RFP-HP1α versus RFP657-HP1α$_{I165E}$ (magenta). **b.** Quantification of the CV$_{index}$ across multiple Hoechst 33342 stained HeLa (HeLa) versus Hela$^{HP1α\text{-}KO}$ nuclei that are not transiently transfected (KO) versus are transfected with RFP657HP1α (KO + HP1α) or RFP657-HP1α$_{I165E}$ (KO + HP1α$_{I165E}$) ($N \geq 28$ cells, three biological replicates). The box and whisker plots in (b) show the minimum, maximum, sample mean: ns $P > 0.05$, **** $P < 0.0001$, one-way ANOVA.

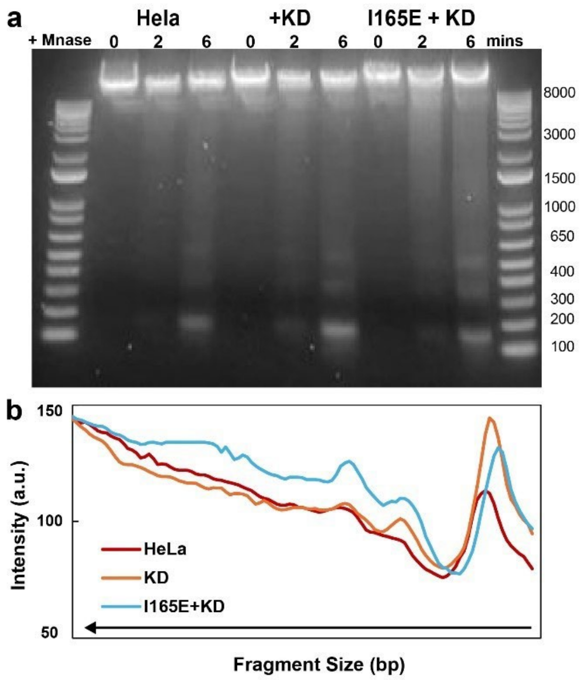

Supplementary Figure 11. Quantification of the role of HP1α monomers and or dimers play in regulating chromatin access by MNase digestion. **(a)** DNA fragmentation product (2 μg in each case) from MNase digestion of HeLa, $HeLa^{KD}$, and $HeLa^{HP1\alpha I165E+KD}$ chromatin for an increasing amount of time (0 min, 2 min, and 6 min at 0.5-unit MNase). **(b)** Quantification of the mean fluorescence intensity of the digested DNA fragments under the 6 min condition.

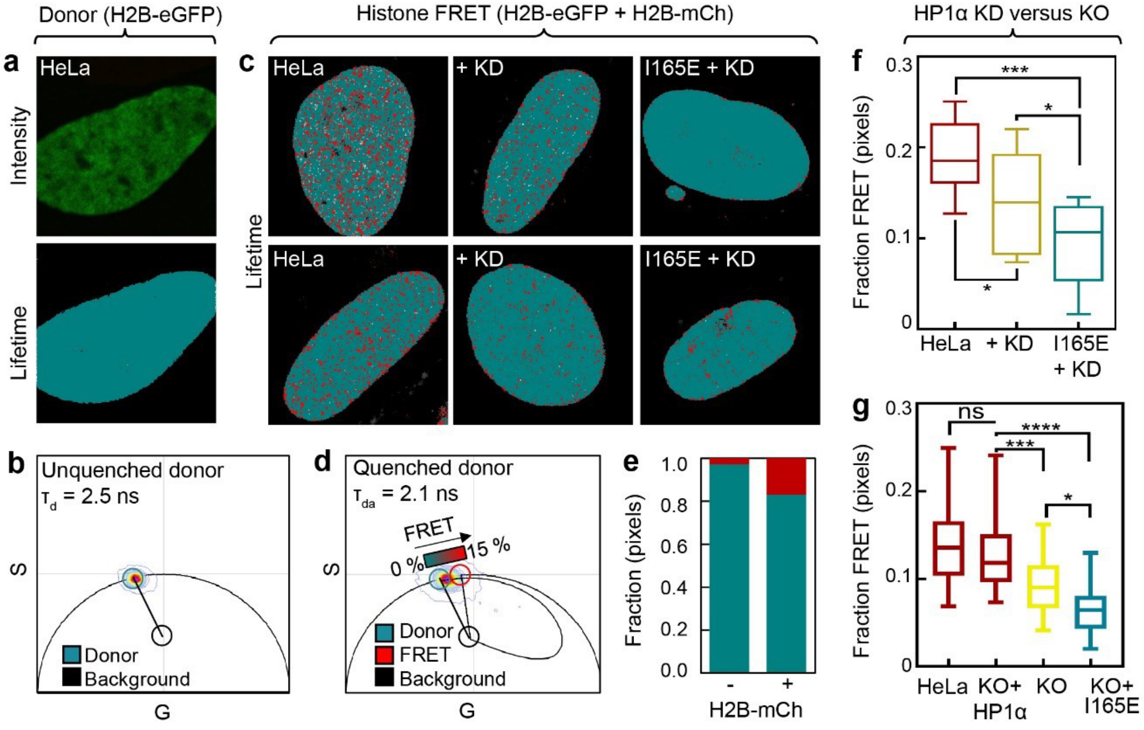

Supplementary Figure 12. Fluorescence lifetime imaging microscopy (FLIM) of histone FRET measures the impact of HP1α self-association on nucleosome proximity in a living cell. (a-b) Intensity image of a HeLa nucleus expressing H2B-eGFP (top) alongside a lifetime map from a FLIM data acquisition of this cell (a) that is pseudo-coloured according to the palette defined in the phasor plot (b), which reveals the unquenched fluorescence lifetime of H2B-eGFP to be 2.5 ns. (**c-e)** Multiple lifetime maps derived from FLIM data acquisitions in HeLa (left), $HeLa^{KD}$ (middle), and $HeLa^{I165E+KD}$ (right) nuclei co-expressing H2B-eGFP and H2B-mCh (c) pseudo-coloured according to a FRET trajectory extrapolated from the linear combination of unquenched donor and background cellular autofluorescence (teal-black) (d) where histone FRET results in a quenched fluorescence lifetime of 2.1 ns and this corresponds to a FRET efficiency of 15 % (e). **(f-g)** Comparison of the impact HP1α self-association has on nucleosome proximity as detected by histone FRET in HeLa versus $HeLa^{KD}$ and $HeLa^{I165E+KD}$ (i.e., knock down (KD) conditions) (f) as well as HeLa versus $HeLa^{KO}$ (KO), $HeLa^{KO+HP1\alpha}$ (KO + HP1α) and $HeLa^{KO+I165E}$ (KO + I165E) (i.e., knock out (KO) conditions) transiently transfected with H2B-eGFP and H2BmCh. The box and whisker plots in (f-g) show the minimum, maximum, sample mean; ns $P > 0.05$, * $P < 0.05$, ** $P < 0.01$, **** $P < 0.0001$, one-way ANOVA.

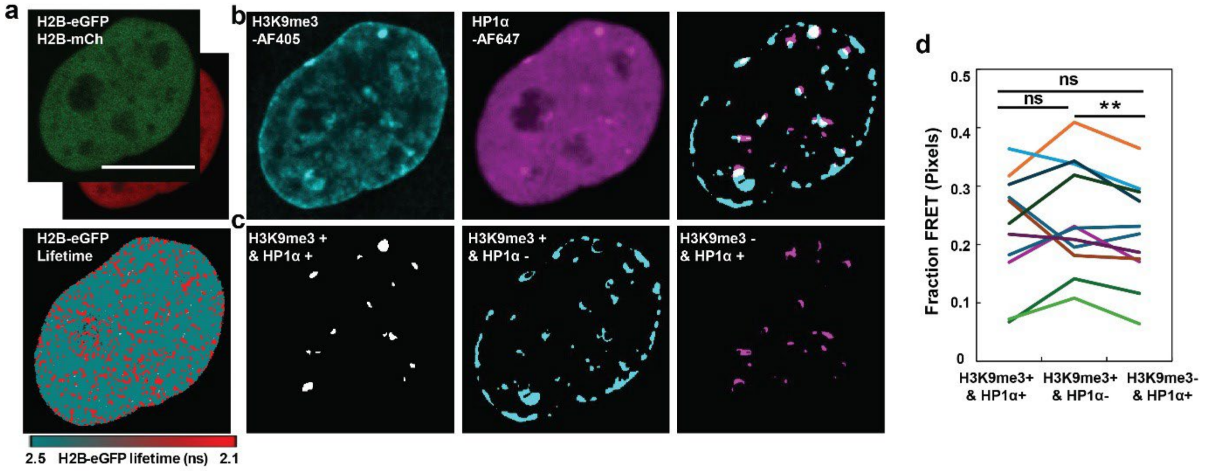

Supplementary Figure 13. H3K9me3 positive chromatin is more compact than HP1α positive chromatin region. (a) Intensity images of a HeLa nucleus co-expressing H2B-eGFP and H2B-mCh (top) alongside the corresponding lifetime map of a FLIM data acquisition pseudo-coloured according to no FRET (teal) versus FRET (red) (bottom). Scale bar 5 µm. **(b)** Immunofluorescence (IF) against endogenous H3K9me3 (left) and HP1α (middle) in the cell presented in (a) alongside a merged intensity mask indicating high H3K9me3 and HP1α IF signal (right). **(c)** Pixels identified from the mask presented in (b) that contain high H3K9me and HP1α signal (i.e., $H3K9me^{+}$ & $HP1\alpha^{+}$) (left), only high H3K9me3 intensity signal (i.e., $H3K9me^{+}$ & $HP1\alpha^{-}$) (middle), and only high HP1α intensity signal (i.e., $H3K9me^{-}$ & $HP1\alpha^{+}$) (right). **(d)** Quantification of the fraction of histone FRET (compact chromatin) within the pixels identified in panel (c): ns $P > 0.05$, ** $P < 0.01$, one-way ANOVA.

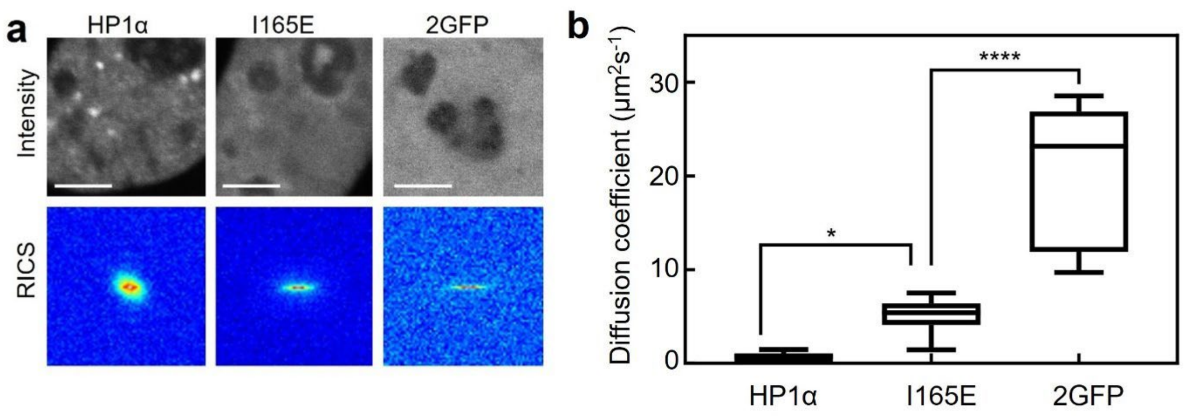

Supplementary Figure 14. **RICS analysis reveals eGFP-HP1α$_{I165E}$ to exhibit a reduced diffusion coefficient in the presence of DNA binding capacity compared to an inert protein of similar size. (a)** Fluorescence fluctuation spectroscopy (FFS) datasets compatible with raster image correlation spectroscopy (RICS) were acquired in selected regions of interest (ROIs) in HeLa nuclei expressing eGFP-HP1α (positive control for chromatin binding), eGFP-HP1α$_{I165E}$ (monomeric HP1α), and 2GFP (negative control for chromatin binding). **(b)** Box plot of the diffusion coefficients extracted from RICS analysis of the FFS datasets presented in (a) serves as a readout of chromatin binding activity (i.e., reduced mobility indicates increased chromatin binding). ($N \geq 6$ cells, two biological replicates). **P*<0.05, *****P*<0.0001, one-way ANOVA.

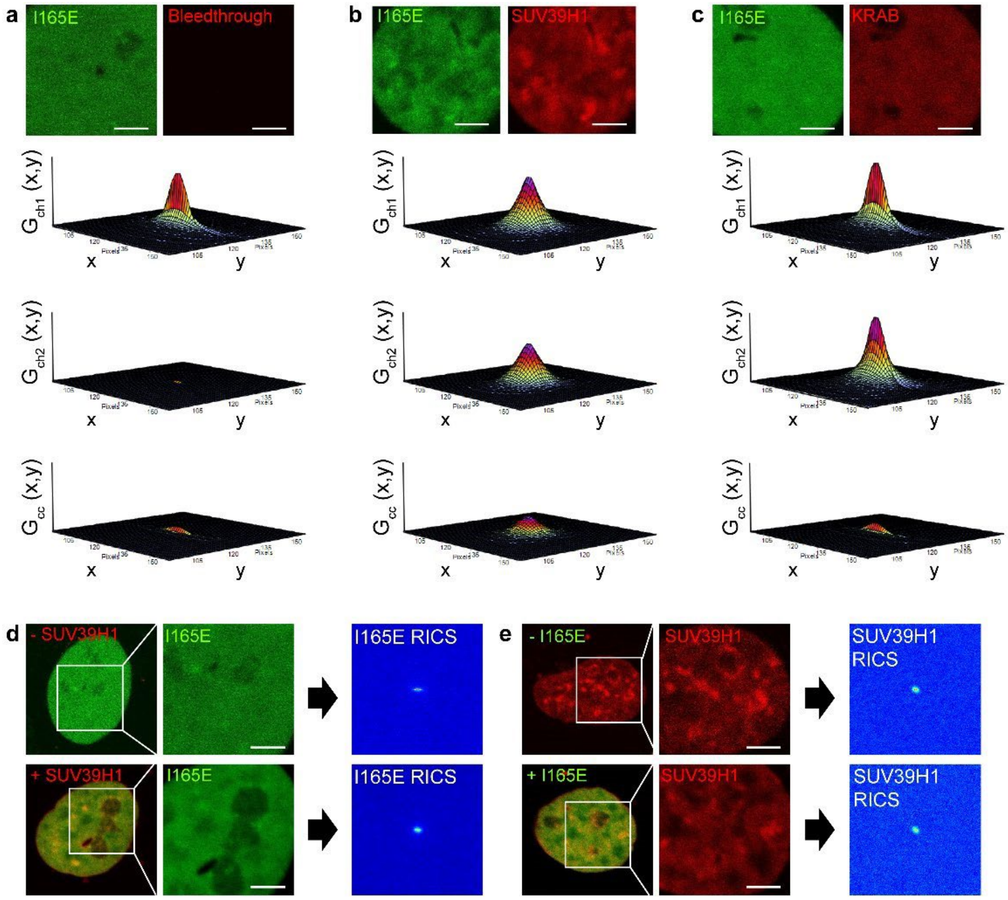

Supplementary Figure 15. RICS and cross RICS analysis of HP1α monomer interaction and mobility. a-c. Intensity images of a region of interest (ROI) selected for a dual-channel frame scan acquisition that is compatible with RICS and cross RICS analysis within HeLa$^{eGFPHP1\alpha I165E+KD}$ nuclei un-transfected (a) versus transiently transfected with SUV39H1-mCH (b) or KRAB-mCh (c) (top row). The RICS versus cross RICS function fits to a 1-component 3D diffusion model are shown below the intensity images. As can be seen in panel (a) (that is a bleed through control) there is negligible cross correlation, while in panel (b) and (c), an example of significant (b) versus insignificant cross correlation (c), reveals eGFP-HP1α_{I165E} to form a heterocomplex with SUV39H1-mCH and not KRAB-mCh. **d.** Merged intensity image of HeLa$^{eGFP\text{-}HP1\alpha I165E+KD}$ nuclei in the absence (top row) versus presence (bottom row) of SUV39H1-mCh transient transfection (left) and the corresponding ROI (middle) selected for RICS (right). As can be seen from a comparison of the shape of the tow RICS profiles presented, the mobility of eGFP-HP1α_{I165E} is significantly slowed down in the presence of SUV39H1mCh; a result confirmed upon fitting these profiles to a 1-component 3D diffusion model in **Fig. 4k**. **e.** Merged intensity image of HeLa^{KD} (top row) versus HeLa$^{eGFP\text{-}HP1\alpha I165E+KD}$ (bottom row) nuclei transiently transfected with SUV39H1-mCh (left) and the corresponding ROI (middle) selected for RICS analysis (right). As can be seen from a comparison of the shape of the two RICS profiles presented, the mobility of SUV39H1-mCh is not significantly slowed down or speeded up in the presence of eGFP-HP1α_{I165E}.

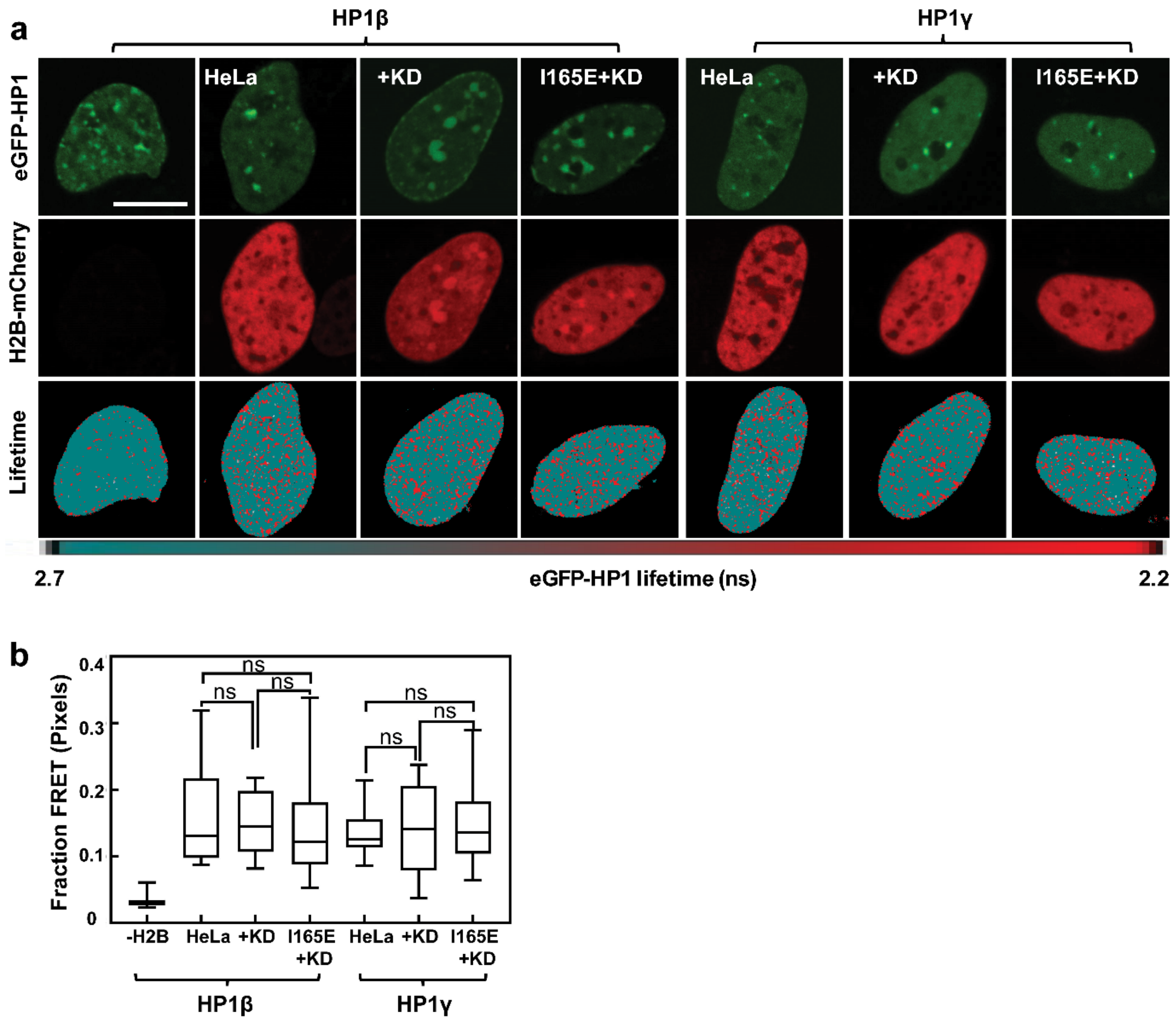

Supplementary Figure 16. FLIM-FRET demonstrates the expression of monomeric HP1α to not impact the capacity of HP1β and HP1γ to bind chromatin. **(a)** Intensity images of eGFP-HP1β versus eGFP-HP1γ in HeLa, HeLa^{KD} and HeLa$^{HP1\alpha I165E+KD}$ nuclei (top row) in the absence versus presence of H2B-mCh (middle row), alongside corresponding FLIM maps (bottom row) pseudo-coloured to spatially map HP1β and HP1γ FRET interaction with H2B (red pixels) (i.e., chromatin binding). **(b)** Quantification of the fraction of HPβ and HP1γ chromatin binding across multiple cells ($N \geq 10$ cells, two biological replicates). The box and whisker plot shows the minimum, maximum, and sample mean: ns $P > 0.05$, one-way ANOVA.

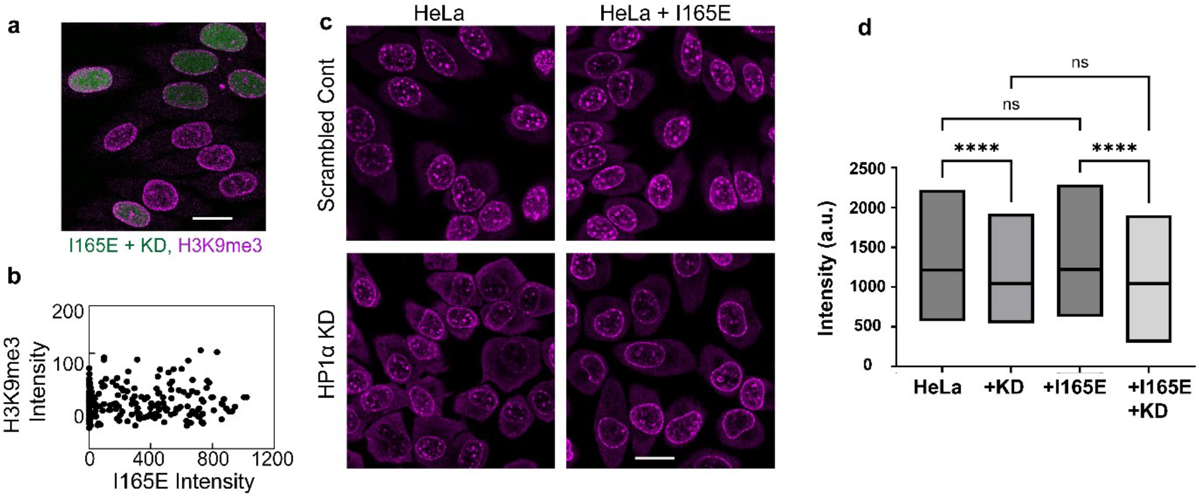

Supplementary Figure 17. HP1α monomer does not affect SUV39H1-mediated H3K9me3. **(a)** Merged intensity image of eGFP-HP1α$_{I165E}$ (green) and H3K9me3 immunofluorescence (IF) labelled with an AF647 tagged secondary antibody (H3K9Me3, magenta) in HeLa$^{eGFP-HP1αI165E+KD}$ cells. Scale bar 20 µm. (**b)** A plot of eGFP-HP1α$_{I165E}$ versus H3K9me3 intensity across multiple nuclei (N = 213 cells, one biological replicate). **(c)** Intensity images of H3K9me3 IF in HeLa and HeLa^{HP1αI165E} nuclei in the absence versus presence of HP1α siRNA. **(d)** Quantification of the mean H3K9me3 IF intensity across multiple HeLa, HeLa^{KD}, HeLa^{HP1αI165E} and HeLa$^{HP1αI165E+KD}$ nuclei (N ≥ 141 cells, two biological replicates). The floating bar plot in (d) shows the minimum, maximum, sample mean: ns $P > 0.05$, **** $P < 0.0001$, one-way ANOVA.
